# RNA-seq analysis of synchronized developing pollen isolated from a single anther

**DOI:** 10.3389/fpls.2023.1121570

**Published:** 2023-04-03

**Authors:** Liam Le Lievre, Sreejith P. Chakkatu, Shiny Varghese, Robert C. Day, Sarah M. Pilkington, Lynette Brownfield

**Affiliations:** ^1^ Biochemistry Department, University of Otago, Dunedin, New Zealand; ^2^ The New Zealand Institute for Plant and Food Research, Lincoln, New Zealand; ^3^ The New Zealand Institute for Plant and Food Research, Auckland, New Zealand

**Keywords:** microspore, pollen, lysis, transcriptome, RNA-sequencing

## Abstract

Pollen development, from unicellular microspores to anthesis, is a complex process involving the coordinated specification, differentiation and functions of different cell types. Key to understanding this development is identifying the genes expressed at precise stages of development. However, transcriptomic studies on pollen prior to anthesis are complicated by the inaccessible nature of pollen developing in the anther and the resistant pollen wall. To assist with understanding gene expression during pollen development we have developed a protocol to perform RNA-Seq on pollen isolated from a single anther (SA RNA-Seq). The protocol involves removing pollen from a single anther for analysis and viewing the remaining pollen to determine the developmental stage. The isolated pollen is chemically lysed and mRNA isolated from the lysate using an oligo-dT column before library preparation. Here, we report on the development and testing of our method and the generation of a transcriptome for three stages of pollen development from Arabidopsis (*Arabidopsis thaliana*) and two stages from male kiwifruit (*Actinidia chinensis*). This protocol enables the transcriptome of precise developmental stages of pollen to be analyzed, and uses a small number of plants, potentially facilitating studies that require a range of treatments or the analysis of the first generation of transgenic plants.

## Introduction

Pollen (male gametophyte) development is a critical step in the life cycle of flowering plants and is essential for plant breeding and seed production. Pollen development occurs within anther locules and is supported by the surrounding anther tissues including the tapetum (reviewed in McCormick, 2004; Borg et al., 2009; Hafidh et al., 2015). Pollen development begins when microsporocytes, or pollen mother cells, undergo meiosis to produce a tetrad of haploid microspores. Following degradation of the microsporocyte wall, each microspore is released into the anther locule. The microspores then increase in size and the pollen wall is deposited. A large vacuole forms and the microspore nucleus migrates to one side producing a polarised microspore. Each microspore then undergoes an asymmetric mitotic division, referred to as pollen mitosis I (PMI), producing bicellular pollen containing two very different daughter cells. The larger vegetative cell exits the cell cycle, plays a supportive role during pollen development and will produce a pollen tube following pollination. The smaller generative cell (representing the male germline) is initially located on the edge of the vegetative cell before being engulfed within the cytoplasm of the vegetative cell to form a cell within a cell structure. The generative cell undergoes a single mitotic division, called pollen mitosis II (PMII), to form the two sperm cells required for double fertilisation.

In Arabidopsis (*Arabidopsis thaliana*) PMII occurs before anthesis with pollen being in a tricellular state when released at anthesis (Borg et al., 2009). However, most angiosperm species (70%) release bicellular pollen, with PMII occurring during growth of the pollen tube (Brewbaker, 1967). The advantage of this reproductive strategy is unknown, although tricellular pollen has been associated with faster germination rate and stricter germination conditions, so this pollination syndrome is more often observed in herbaceous annuals or weeds which favour rapid reproductive cycles (Heslop-Harrison, 1987; Williams et al., 2014). In contrast, bicellular pollen is longer-lived and is often dormant when dispersed, making it advantageous in uncertain pollination environments.

The development of pollen from released uninucleate microspores to anthesis, is thus a complex process involving the coordinated specification, differentiation and functions of several different cell types. This process can be better understood through identification of the genes expressed at precise stages of development, how these genes are regulated, and how environmental conditions alter gene expression. Transcriptomic analyses in Arabidopsis have revealed a developmental shift from an early proliferative phase to a later differentiation phase (reviewed in Rutley and Twell, 2015; Klodová et al., 2022). The early phase includes the microspores and bicellular pollen and is defined by a greater number of expressed genes with a wider overlap with sporophytic genes along with protein production. The late phase is defined by a reduction in the number of expressed genes along with an increase in pollen-enriched and pollen-specific genes along with vesicle transport, pH regulation and energy metabolism.

However, transcriptomic studies on pollen prior to anthesis are complicated by the inaccessible nature of developing pollen within the anther and the resistant pollen wall that is deposited early in development. A key challenge is the isolation and separation of developmental stages. In Arabidopsis, Fluorescence-Activated Cell Sorting (FACS) can be used to separate microspores from other developmental stages based on their small size and autofluorescence levels, but this method cannot separate the bicellular and tricellular stages and requires specialised equipment (Borges et al., 2012). A density gradient centrifugation that allows for the separation of all developmental stages based on size and weight was also developed for Arabidopsis and has been used in several studies (Honys and Twell, 2004; Dupl’áková et al., 2016; Klodová et al., 2022). However, due to size variation, each fraction typically contains 75 to 85% of the target stage, and pollen across the entire developmental stage is represented (Dupl’áková et al., 2016). Thus, the isolated fractions represent a developmental window rather than a precise stage. Gradient centrifugation is also time-consuming, with stage separation requiring 2 to 2.5 h following the harvesting of inflorescences, leading to potential transcriptional changes during isolation (even though the material is kept cool). Moreover, this protocol is difficult to scale up to study a range of different lines or treatments. In rice, laser capture microdissection has been used to separate different stages of developing pollen (Hobo et al., 2008; Suwabe et al., 2008), although this required 250-400 dissections using specialized equipment. Other studies have relied on floret morphology in rice (Wei et al., 2010), or bud size in tobacco (Bokvaj et al., 2015) and tomato (Keller et al., 2018) to separate developmental stages. However, like density centrifugation this provides a pollen sample containing a developmental period rather than a precise stage. In a recent study, Nelms and Walbot (2022) used single pollen grains from maize and aspirated pollen contents to perform RNA-Seq, which avoids the need to separate stages but is likely difficult in species with smaller pollen grains such as Arabidopsis.

To facilitate future transcriptomic studies of pollen during development in the anther we developed a protocol to enable RNA-Seq analysis of pollen isolated from a single anther which could be used in a range of species. Using a single anther provides several advantages. As pollen within a single anther is largely developmentally synchronized (Zhang et al., 2002), the transcriptomic data generated by such an approach will relate to a precise developmental stage, aiding studies exploring different stages. To ensure such comparisons can be made, we also wanted to enable the developmental stage of the analyzed pollen to be determined by microscopy, rather than through a proxy measurement such as bud size. Further, using a single anther rather than bulk-harvested pollen means that only a single or a few plants are required to provide multiple samples, including biological replicates. This offers advantages for comparative transcriptomic studies, such as studies assessing the impact of a range of environmental stimuli (e.g., different temperatures) or using multiple Arabidopsis accessions. Further, using a single anther enables the analysis of the first generation of transgenic plants when investigating the impact of overexpressing or knocking-out putative transcriptional regulators or other proteins in transgenic plants. We also wanted to develop a protocol that could be performed without the need for highly specialised equipment and could be easily adapted to non-model plant species. Here we describe the development of our protocol for RNA-Seq on pollen from a single anther and present our analysis for pollen stages from Arabidopsis and kiwifruit.

## Materials and methods

### Plant material and growth conditions

*Arabidopsis thaliana* Col-0 plants were grown at 20-21°C under a 16 h light and 8 h dark cycle, with approximately 70% relative humidity.

All kiwifruit samples were from the male kiwifruit cultivar *Actinidia chinensis* var. *chinensis* acc. ‘Russell”. Canes were collected from mature (at least 6-year-old) kiwifruit vines from the Plant & Food Research orchard in Motueka, New Zealand (41° 9’ S, 172° 98’ E), in late autumn. Canes were stored in the dark at 4°C for at least one month before floral induction. Canes with at least 10 mm basal diameter were cut to approximately 15 cm in length and the proximal ends were placed in 100 ml autoclaved distilled water. The water was continually aerated and changed regularly to prevent infection or build-up of organic exudate. Canes were incubated at 20°C with a 16-h-light and 8-h-dark cycle until floral buds were harvested.

### Pollen isolation and stage determination

#### Arabidopsis

Healthy primary inflorescences were removed from a plant once at least four flowers had opened. The inflorescence, with open flowers removed, was placed in distilled water on a clean microscope slide under a Nikon SMZ800 dissecting microscope. Buds were removed in order from the most mature bud (-1 bud) to approximately the -12 bud using fine forceps (Dumont #55, 0.05 x 0.02mm tip). The buds were placed in water on a clean microscope slide and remained wet on the slide until pollen isolation. A selected bud was transferred to a new clean microscope slide and placed in a drop of distilled water. Using a sharp needle (gauge size 27G ½”), the sepals and petals were removed or opened (depending on bud age). A single medial (long) anther was isolated by cutting the filament with the needle. This anther was transferred to a 2 to 3 µl drop of sterile 0.3 M D-mannitol (Sigma Aldrich), on a clean microscope slide. The anther was then cut open using the needle releasing 50 to 100 pollen grains into the mannitol. Further mannitol (7-8 µl) was added and the pollen/mannitol mix (approximately 10 µl) transferred by pipette to a 200 µl thin-walled PCR tube. The pollen mix was centrifuged for 2 min in a fixed speed minicentrifuge (Gilson™ GmCLab Microcentrifuge; approx. 2000 g). The supernatant was aspirated, and the pollen resuspended in 10 µl of fresh 0.3 M D-mannitol, and the wash repeated. After a third centrifugation, the mannitol was removed. The pollen sample was either resuspended in DAPI (4’-6-Diamidino-2-phenylindole; 10 µg.mL^-1^ DAPI, 0.1 M sodium phosphate, pH 7; 1 mM EDTA, 0.1% (v/v) Triton X-100) and viewed under an Olympus BX51 upright fluorescence microscope with a U-MWU2 filter set (excitation 330-385 nm and emission 420 nm LP) or snap frozen in liquid nitrogen and stored at -80 °C until lysis.

The dissected anther material and remaining pollen was transferred to a 200 µl tube containing 100 µl Farmer’s solution (3:1 v/v ethanol: acetic acid). The material was incubated for at least 4 h at room temperature and stored at 4°C until use. To determine developmental stage the anther material was removed from the fixative using fine forceps and placed in a clean microscope slide. The anther was rinsed in 3 µl of water for 5 sec and then placed in 3 µl of DAPI. The anther was dissected further using the needles to release the remaining pollen. A further 7-10 µl of DAPI was added, the pollen incubated for 10 min and the material covered with a coverslip and sealed with nail varnish. The DAPI-stained pollen was viewed under an Olympus BX51 upright fluorescence microscope as described above within 2-3 hours of DAPI treatment and images acquired using Nikon NIS Elements AR software.

#### Kiwifruit

To determine the bud size from which pollen at specific stages can be isolated, 50 buds were measured for height and width with fine calipers, and extracted pollen was analyzed by microscopy to determine pollen developmental stage. The height of buds was determined by fine measurement from the base of the receptacle to the top of the bud, and width was determined by a transverse measurement in the middle of the bud at its thinnest point. Buds were cut in half longitudinally and anthers were removed with forceps and placed in a drop of water on a microscope slide. Using a sharp needle (gauge size 27G ½”), a small nick was made in a single locule to release contents. A small volume of water (~2-3 µL) containing at least 100 developing pollen cells was transferred to a 200 µL PCR tube containing 20 µL of Farmer’s solution. The pollen samples in Farmer’s solution were centrifuged for 30 seconds in a minicentrifuge (approx. 2000 g), and excess solution careful aspirated. The pellet was resuspended in ~20 µL of DAPI solution and transferred onto a clean microscope slide. A clean cover slip was placed on the droplet, which was sealed using nail varnish and viewed with an upright fluorescence microscope as for Arabidopsis pollen.

For the isolation of pollen for RNA-Seq, whole buds of ~6 mm diameter and 4.5 mm height (the dimensions shown to indicate uninucleate microspores and bicellular pollen) were excised from canes and sliced in half longitudinally under a light microscope. Intact single anthers were then carefully extracted with forceps and placed on a clean microscope slide and immersed in ~20 µL of 0.3 M D-mannitol. The distal end of the anther lobes was sliced open using two clean 1.6 x 40 mm microlances (Becton, Dickinson and Company), releasing pollen into the solution. A small sub-sample (~2-3 µL) of the solution containing at least 100 pollen grains was pipetted into 200 µL volume strip-cap tubes containing 20 µL Farmer’s solution for microscopic determination of developmental stage. The remaining reproductive cells were moved into a separate 200 µL PCR tube and washed using mannitol as described for Arabidopsis pollen.

To determine stage, the pollen samples in Farmer’s solution were briefly centrifuged (approx. 2000 g), and excess solution aspirated. The pellet was resuspended in ~10 µL of DAPI solution, then transferred onto a clean microscope slide. A clean cover slip was placed on the droplet, which was sealed using nail varnish and viewed as for Arabidopsis pollen.

### Pollen lysis

Pollen lysis conditions were investigated on pollen isolated from -1 Arabidopsis buds as described above, but the pollen was released into water rather than mannitol. The pollen was transferred to 10 µl of a 4M 4-Methylmorpholine *N*-oxide monohydrate (NMMO; Sigma Aldrich) solution that was pre-warmed on a clean microscope slide placed on a controlled-temperature heating block. During the incubation additional water was added to keep the volume approximately constant as some evaporation occurred. Following the incubation, a cover slip was placed on top of the pollen and pollen viewed by brightfield microscopy using an Olympus BX51 upright fluorescence microscope. The percentage of pollen lysis was determined by viewing 100 pollen grains and observing if the sporoplast was expelled from the pollen wall. The experiment was repeated on three occasions for each incubation condition.

For RNA-Seq analysis, the washed Arabidopsis or kiwifruit pollen samples were allowed to thaw on ice and 3.5 µl (Arabidopsis) or 5 µl (kiwifruit) of a 4 M NMMO solution containing 2U/ml RNaseOUT (Thermo Fisher Scientific) was added. The mix was briefly vortexed before being incubated in a pre-heated thermocycler (Eppendorf Flexilid) for 10 min at 75°C. The mix was vortexed for 5 sec and centrifuged for 5 min at approx. 2000 g to remove unlysed pollen along with wall and cellular debris. We refer to the supernatant as pollen lysate.

### Measuring the impact of NMMO on cDNA synthesis

To determine if the pollen lysis conditions impacted RNA integrity, RNA was purified from 100 mg of Arabidopsis leaf material or young kiwifruit leaf material. The leaf material was frozen in liquid nitrogen and ground into a fine powder with a mortar and pestle and RNA isolated using an RNeasy Plant Mini Kit (Qiagen, Germany) following the manufacturer’s instructions. Total RNA was eluted in 50 µL of ultra-pure water (Thermo Fisher Scientific), and only RNA with an integrity and quality (IQ) score of ≥ 9.5, as determined with a Qubit RNA IQ assay kit (Thermo Fisher Scientific), was used. For the Arabidopsis leaf RNA, ~1 ng of RNA was either incubated on ice (control) or under pollen lysis conditions, before the RNA integrity was determined using a 2100 BioAnalyzer Eukaryote Total RNA Pico RNA Chip (Agilent Technologies). For the kiwifruit leaf RNA, 1 µg of the RNA was incubated under lysis conditions or on ice (control) for ten minutes and then converted to cDNA with SuperScript II Reverse Transcriptase (Invitrogen) according to the manufacturer’s instructions. This cDNA was then purified using AMPure XP beads to remove NMMO, and 10 ng was run on an HS-DNA BioAnalyser chip (Agilent Technologies).

### Testing for anther-derived transcripts

Pollen was isolated into 0.3 M mannitol from -9 Arabidopsis buds as described above. Following transfer to the PCR tube, the volume of mannitol was adjusted to ~12 μl. One pollen sample was centrifuged once, and the supernatant and the pollen were frozen separately as the sample’s supernatant 1 and pellet 1. Another pollen sample was centrifuged three times with the supernatants from the second and third centrifugation and the final pellet frozen separately giving samples supernatant 2, supernatant 3 and pellet 3. Each sample was split into two 5 μl aliquots and cDNA prepared using an oligo(dT) primer and Superscript III Reverse Transcriptase (RT; Invitrogen) from one aliquot and the other aliquot used as a no RT control. Quantitative PCR used 1 µl of the +/- RT samples, primers for the tapetal-expressed gene *LTP12* (*At3g51590* F; 5’ TGGCATCCCCAACAGAGTCA and R; TGGCATCCCCAACAGAGTCA; Xu et al., 2010) or the constitutive *Actin 2* (*At3g18780* F; 5’ CGCTCTTTCTTTCCAAGCTCAT and R; 5’ TCCTGCAAATCCAGCCTTC) and the qPCRBIO SyGreen Mix (PCRBiosystems) with primer efficiencies determined to be between 0.9 and 1.1 by serial dilutions of the PCR product. Reactions were run on a Lightcycler480 with a 96 Block (Roche).

### Preparation of cDNA for RNA-sequencing

cDNA libraries were prepared in a two-stage process whereby the polyadenylated RNA was reverse transcribed to cDNA using an oligo-dT primer in the presence of a template switching oligo (TSO; Macaulay et al., 2016), followed by a PCR preamplification giving amplified cDNA (acDNA). Sequencing libraries were prepared either directly from pollen lysate or following mRNA enrichment. For cDNA libraries produced directly from the pollen lysate the protocol in Tantirigama et al. (2016) was followed but without additional Mg^2+^ being added at the cDNA synthesis stage, as in a preliminary experiment the addition of Mg^2+^ lead to amplification from rRNA. For the mRNA enriched material, cDNA synthesis and amplification were performed on the beads according to Macaulay et al. (2016) except the concentration of RNaseOUT (Invitrogen) used in all relevant solutions was adjusted to 2 U.mL^-1^ (the working concentration recommended in manufacturer instructions). To remove enzymes, salts and other contaminants the amplification products were purified using 12.5 µL of AMPure XP beads (Beckman Coulter) following the manufacturer’s instructions, except that a 1:1 ratio of acDNA solution:AMPure XP bead solution was used as in Macaulay et al. (2016). The quality of the acDNA was determined by inspection of electropherograms generated using a High-Sensitivity (HS) DNA Bioanalyzer chip (Agilent Technologies) with a unimodal peak of acDNA fragments around 1.3 kbp indicating high-quality samples.

### Library preparation and sequencing

A Nextera XT DNA Library preparation kit and Nextera XT 96-index kit was used to prepare a total of 32 Illumina-compatible sequencing libraries across three experiments from both the direct lysate-derived acDNA and mRNA-enriched acDNA according to manufacturer’s instructions (Illumina), except the final volume was adjusted to 25 µL rather than 50 µL. The concentration of Nextera libraries were determined using a Qubit dsDNA High-Sensitivity (HS) kit (Thermo Fisher Scientific) run on a Qubit 2.0 fluorimeter (Thermo Fisher Scientific), and average fragment length was calculated using 2100 Bioanalyzer (Agilent Technologies) or Fragment Analyser (Agilent Technologies) reports and libraries were normalised by dilution to 4 nM.

Sequencing of all 32 libraries (**Supplementary Table 1**) was performed on a HiSeq2500 (Illumina) at the Otago Genomics Facility, housed in the Department of Biochemistry at University of Otago, Dunedin, NZ. Each experiment (**Supplementary Table 1**) was sequenced at a different time. Arabidopsis samples were run across two lanes with a 2x150bp paired-end V2 Rapid sequencing kit and kiwifruit samples were run across five lanes with a 2x250bp paired-end V2 Rapid sequencing kit.

### Analysis & data availability

Raw sequence data generated in this study is available through the NCBI Sequence Read Archive (SRA); ID number SUB12698543; Bioproject PRJNA930874. To compare to existing RNA-seq data of bulk isolated pollen in Arabidopsis (Col-0), three samples of raw sequencing data generated by the EVOREPRO consortium (ArrayExpress Accession ID E-MTAB-9456; Julca et al., 2021; Klodová et al., 2022) was downloaded for each stage of: uninucleate microspores, late bicellular pollen, and tricellular pollen. Gene expression levels was determined for this data in the same manner as the data generated in this work.

Raw sequence data was filtered for adapter sequences and poor-quality reads using *fastp* v0.22.0 (Chen et al., 2018) with default parameters for both Arabidopsis and kiwifruit. However, for the kiwifruit data which utilised 250 bp chemistry –include_unmerged was also selected, so that unpaired or orphaned reads from read pairs with insert sizes shorter than the 250 bp were included in downstream analyses. This inclusion did not incur significant penalty to the metrics for aligned sequences. Filtered reads were then aligned to the reference genome using STAR v2.7.10a with default parameters except for the –seedPerWindowNmax which was set to 30. The Arabidopsis TAIR10.1 (BioProject: PRJNA10719) or the male *A. chinensis* acc. ‘Russell’ genome (assembly and annotation files available online at https://figshare.com/s/c6ffcc5300533022cc5a; Tahir et al., 2022) genome assemblies were used as appropriate. The tool featureCounts from the Subread 2.0.3 package (Liao et al., 2014) was used with default parameters to determine expression level of genes for each sample, based on the feature annotations from the Araport11 gene model assembly (Cheng et al., 2017) for Arabidopsis and the Russell gene models for kiwifruit (Tahir et al., 2022). Quality control package Qualimap v2.2.2 (García-Alcalde et al., 2012) was used to determine number of genomic alignments which fall into exonic, intronic or intergenic regions for the Arabidopsis data generated in this study. For this analysis Qualimap v2.2.2 was set to utilise paired end reads and include multi-mapped reads. While multi-mapped reads are not usually included when performing read mapping and quantification, we included them as we were only assigning reads to one of three categories; exon, intron or intergenic rather than determining read counts per individual gene. Incorporation of multi-mapped reads using Qualimap is proportional; one read mapping to four sites assigns 0.25 read counts to each gene so that this read is not over-represented relative to uniquely mapped reads.

To determine genes considered ‘expressed’ in each developmental stage, genes were filtered for those that had TPM ≥ 2 in at least two samples. This threshold was chosen to more appropriately compare gene expression in the low-input, lower diversity single anther RNA-seq samples to the higher-input, more sensitive bulk isolated pollen samples described in Klodová et al., 2022. Venn diagrams illustrating genes expressed in pairwise comparisons across stages and RNA-seq protocols were generated using Venny (Oliveros, 2015). Differential expression analyses between stages and RNA-seq protocols were then performed using DESeq2, with a false discovery rate-adjusted p-value (FDR) of <0.01 for the detection of differentially expressed genes (DEGs). Because there were differences in library sizes across samples (**Supplementary Table 1**), expression data used for differential expression analysis was normalised using the *varianceStabilizingTransformation* function from DESeq2. Principal component analysis (PCA) plots and hierarchical clustering was performed on the variance stabilised data using the *plotPCA* and *hclust* functions from the DESeq2 and R stats packages, respectively.

To construct a common proxy for comparative analyses between Arabidopsis and kiwifruit, peptide sequences for both assemblies were used to identify Reciprocal Best Hits (RBHs). BLASTp was run for each Arabidopsis peptide sequence against the *A. chinensis* peptide sequences with an e-value cut-off of 1e-3, and *vice versa* for *A chinensis* peptides against Arabidopsis peptide sequences. For pairwise Venn diagram and differential expression analyses across different species, only genes for which an RBH was identified were used.

To test the significance of overlap of expressed genes between samples from different RNA-seq protocols, tissue types, or species, an Odds Ratio calculated from a Fisher’s exact test was generated using the *GeneOverlap* R package. When comparing across species, the list of RBHs (12465) was used as the “genome size”. Leaf tissue expression data from Berkowitz et al. (2021); data taken from **Supplementary Table 1** with TPM >2) was used as a negative control to test whether data generated in the current study had higher similarity to existing pollen data compared to other tissue types. A total of 16243 genes (containing 9018 RBHs) from the leaf data showed expression of TPM ≥ 2, which was used as the comparison to SA RNA-seq libraries, against a total TAIR10 “genome size” of 32894 genes.

Gene ontology enrichment analyses were performed on DEGs using ShinyGO v0.76.3 (Ge et al., 2020) and presented using the ggplot2 R package as described in Bonnot et al. (2019). For kiwifruit DEG analysis, the TAIR ID for RBHs were used to determine enriched GO terms.

## Results and discussion

### Isolation of pollen at specific developmental stages from Arabidopsis

**Figure 1:**
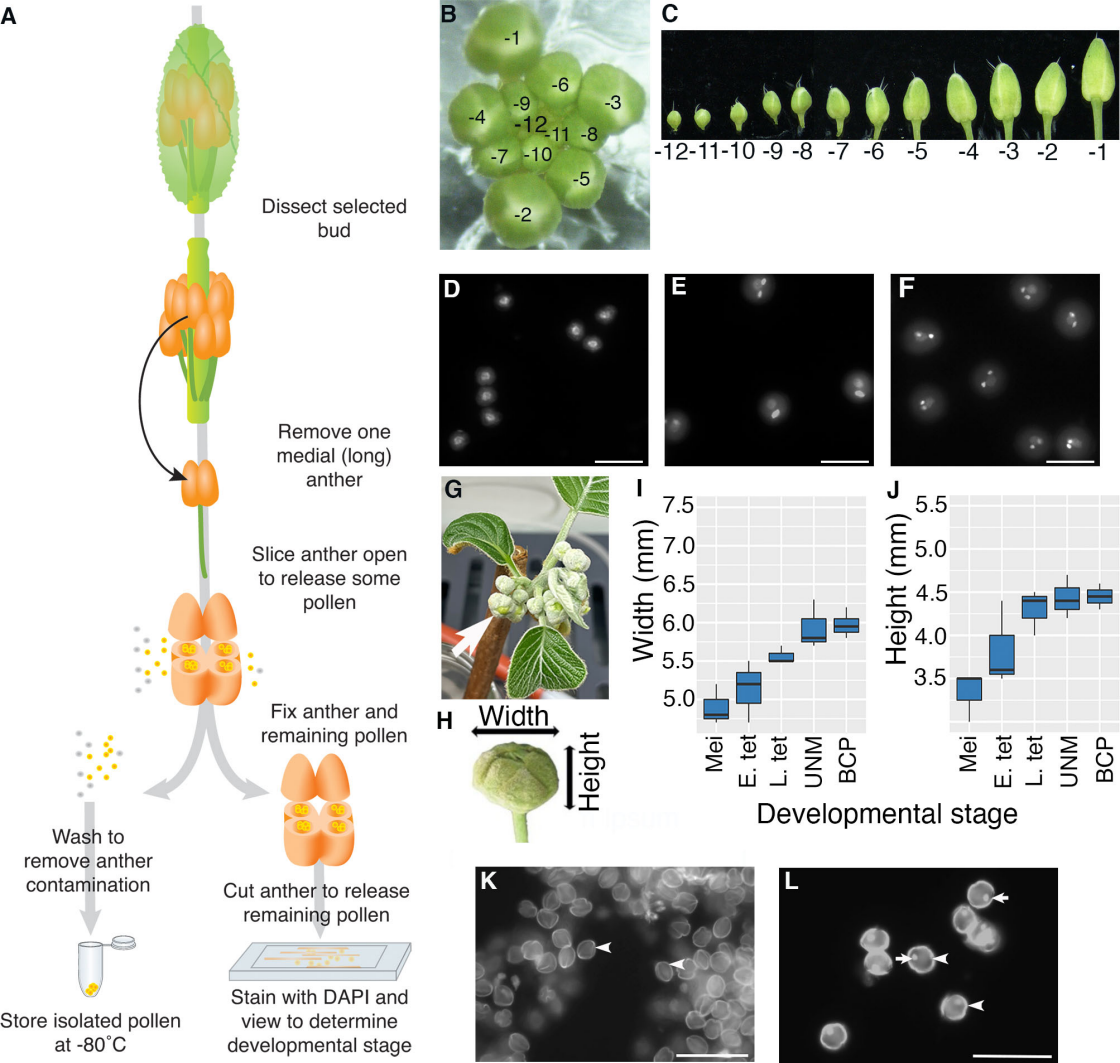
Isolation of pollen from a single anther. **(A–F)** Arabidopsis and **(G–L)** kiwifruit. **(A)** Flow diagram illustrating how pollen is isolated from a single anther from a bud from an Arabidopsis plant. A majority of the pollen is used for RNA-seq analysis (frozen), and the remaining pollen and anther material is used for determining the stage of pollen development. **(B)** An Arabidopsis inflorescence with the relative age of buds indicated with -1 representing the bud closest to opening, the second unopened bud -2 etc. **(C)** Buds removed from an inflorescence in order from the youngest bud dissected (-12) to the oldest unopened bud (-1). **(D–F)** Representative DAPI-stained pollen from Arabidopsis showing different stages of pollen development; uninucleate microspores after release from the tetrad with central nuclei **(D)**, pollen close to pollen mitosis II with a mix of late bicellular pollen with an elongated generative cell nucleus and some tricellular pollen **(E)** and tricellular pollen with two brightly stained sperm cells **(F)**. Images are not all from the same inflorescence. Scale bars = 20 μm. **(G)** A kiwifruit node from a male plant with developing inflorescences in an indoor growth facility. The node has several cymes each with a terminal flower (arrow) and lateral buds. **(H)** An isolated terminal bud from a kiwifruit inflorescence indicating the width and height. **(I, J)** Terminal buds removed from kiwifruit nodes were measured for width **(I)** and height **(J)** before pollen mother cells/pollen were removed to determine the developmental stage. Box and whisker plots show the median and interquartile range of 10 buds for each developmental stage. Mei: meiotic cells; E. tet, early tetrads; L. tet, late tetrads; UNM, uninucleate microspores; BCP, bicellular pollen **(K, L)** Representative DAPI-stained pollen from kiwifruit; uninucleate microspores after release from the tetrad with the microspore wall showing bright autofluorescence (arrowhead) **(K)** and bicellular pollen with wall autofluorescence (arrowhead) a brightly stained generative cell (arrow) **(L)**. In kiwifruit pollen the microspore (UNM) and vegetative cell (BCP) nuclei are weakly stained by DAPI and cannot be easily seen. Scale bars = 500 μm.

Our aim was to perform RNA-Seq on pollen from a single anther as it is easy to harvest and provides a developmentally synchronized sample. We also wanted samples that are free of contaminating a cell types and are isolated in a manner that limits potential transcriptional changes. In addition, we wanted to be able to determine the developmental stage of the pollen. To achieve this, we used Arabidopsis to develop a protocol that is summarised in **Figure 1A**.

Arabidopsis inflorescences develop in a spiral with the lower, larger buds being the oldest (closest to maturation) and the smaller inner buds being less mature (Smyth et al., 1990). To follow the stage of pollen development we labelled buds based on developmental order with the -1 bud representing the first unopened bud, -2 the second etc. (**Figure 1B**). To begin to isolate pollen, an inflorescence was removed from a healthy Arabidopsis plant, any opened flowers removed, and the inflorescence placed in a drop of water on a microscope slide under a dissecting microscope. We selected primary inflorescences from a raceme with at least four open flowers. Buds were then dissected from the inflorescence and placed in water on a microscope slide in developmental order (**Figure 1C**). We dissected through to the -12 bud which generally contains either tetrads or microspores that have recently been released from tetrads. Buds from inflorescences remained in water on the slide while pollen was being isolated from other buds and water was added as necessary to offset evaporation.

For Arabidopsis pollen isolation a single medial (long) anther was removed from a selected bud by cutting the filament. The anther was placed in a mannitol solution (as an osmo-protectant) on a microscope slide. Under a dissecting microscope, the anther was cut using a sharp needle so the outer wall of several anther locules is torn. This released approximately 50-100 pollen grains into the mannitol solution. The released pollen/mannitol mix was transferred to a tube and washed with mannitol. When the pollen pellet was resuspended in DAPI solution and viewed by fluorescence microscopy, no sporophytic cells or other floral debris were observed (data not shown).

To be able to relate transcriptional changes to developmental stage, we wanted to confirm the developmental stage of the isolated Arabidopsis pollen. To do this, the remaining anther material, along with remnant pollen within the anther, was transferred to another tube and fixed. The fixed anther material was further cut using sharp needles to release the remaining pollen, stained with DAPI and viewed by fluorescence microscopy. We generally observed around 10 to 50 pollen grains, from which different developmental stages could be distinguished based on nuclear morphology (**Figures 1D–F**). Pollen grains from a single anther generally had similar morphology, confirming that pollen within a single anther is largely developmentally synchronized. However, some small differences were observed, most notably close to PMI and PMII where pollen grains that have not yet entered mitosis, mitotic cells and pollen at the following stage of development can all be observed in a single anther (**Figure 1E**). We also found that the same developmental stage could be found in anthers from slightly different bud numbers from different inflorescences/plants (e.g., late bicellular pollen could be in -4 or -3 buds), highlighting the importance of determining the developmental stage for anthers from each bud in an inflorescence.

### Isolation of pollen at specific developmental stages from kiwifruit

To trial the method on a non-model plant we selected kiwifruit, a horticulturally important crop in NZ with different floral morphology to Arabidopsis (Fraser and McNeilage, 2016). As kiwifruit is dioecious, we analysed buds from male plants that produce viable pollen. Budwood bearing dormant inflorescence nodes were cut from canes harvested from orchard-grown plants in autumn and induced to budbreak within indoor growth facilities (**Figure 1G**). Kiwifruit bears inflorescences that are compound dichasium, so each budwood node produces a number of branched cymes each bearing a terminal flower that develops first, and two lateral buds that develop later. For this work only terminal buds were selected (arrow **Figure 1G**). Kiwifruit flowers also contain a greater number of anthers compared to Arabidopsis, with the cultivar used in this work producing 50 to 60 anthers per flower (data not shown). Additionally, kiwifruit pollen is in the bicellular state when released from the anther (Matsunaga et al., 1996).

To get an approximate idea of the appropriate time to harvest kiwifruit buds for pollen isolation, anthers from various sized buds were fixed, stained with DAPI and viewed. This revealed that buds with a width of ~6 mm and height of ~4.5 mm, contained developing pollen (**Figure 1H–J**), although there is some variation in size and no clear difference in size between buds containing uninucleate microspores or bicellular pollen. This differs from other species such as tobacco (*Nicotiana tabacum*), which like kiwifruit releases bicellular pollen, where there is a clear difference in buds containing uninucleate microspores, early bicellular pollen and late bicellular pollen (Bokvaj et al., 2015). Thus, pollen stage in kiwifruit cannot be determined based on size alone. Furthermore, pollen development within the anthers of a single flower is not well synchronised in kiwifruit (data not shown). This highlighted the importance of taking a sample of pollen to determine the developmental stage of the pollen in each anther by microscopy.

The protocol developed for Arabidopsis was adapted for kiwifruit to isolate pollen and determine the stage. A selected kiwifruit bud (~6 mm x 4.5 mm) was removed from the inflorescence, opened and a single anther removed and placed in a drop of mannitol on a microscope slide. The anther was then opened with a sharp needle (as for Arabidopsis), releasing pollen into the mannitol. As anthers from male kiwifruit plants contain a greater number of pollen grains (~4000 per anther) than Arabidopsis, this allowed the released pollen to be used for both transcriptomic analysis and staging. Around 100 pollen grains were transferred to fixative and the remaining pollen was washed and frozen and processed for RNA-Seq.

To determine the developmental stage to the kiwifruit samples the fixed pollen was centrifuged, the fixative solution was replaced with DAPI and the pollen transferred to a clean microscope slide. Unicellular microspores (**Figure 1K**) and bicellular pollen (**Figure 1L**) could be easily distinguished based on the presence or absence of the highly fluorescent smaller generative cell, and pollen isolated from a single anther was largely synchronized.

Overall, this protocol enables the isolation of a pollen sample of a known stage from different species. Furthermore, the pollen is frozen within eight minutes from selection of the bud, limiting transcriptional changes during the isolation process. This compares favourably with previous density centrifugation approaches to harvest pollen which involves lengthy grinding, filtering and centrifugation steps (Honys and Twell, 2004; Dupl’áková et al., 2016). The protocol also enables isolation of a precise developmental stage, unlike bulk harvested pollen which contains a developmental window that is enriched for a stage (Honys and Twell, 2004; Wei et al., 2010; Bokvaj et al., 2015; Dupl’áková et al., 2016; Keller et al., 2018). Having precise developmental stages will help the study of genes involved in processes such as cell-cycle control, microspore polarisation and other genes which may show developmental-stage specific expression.

### Pollen lysis

**Figure 2:**
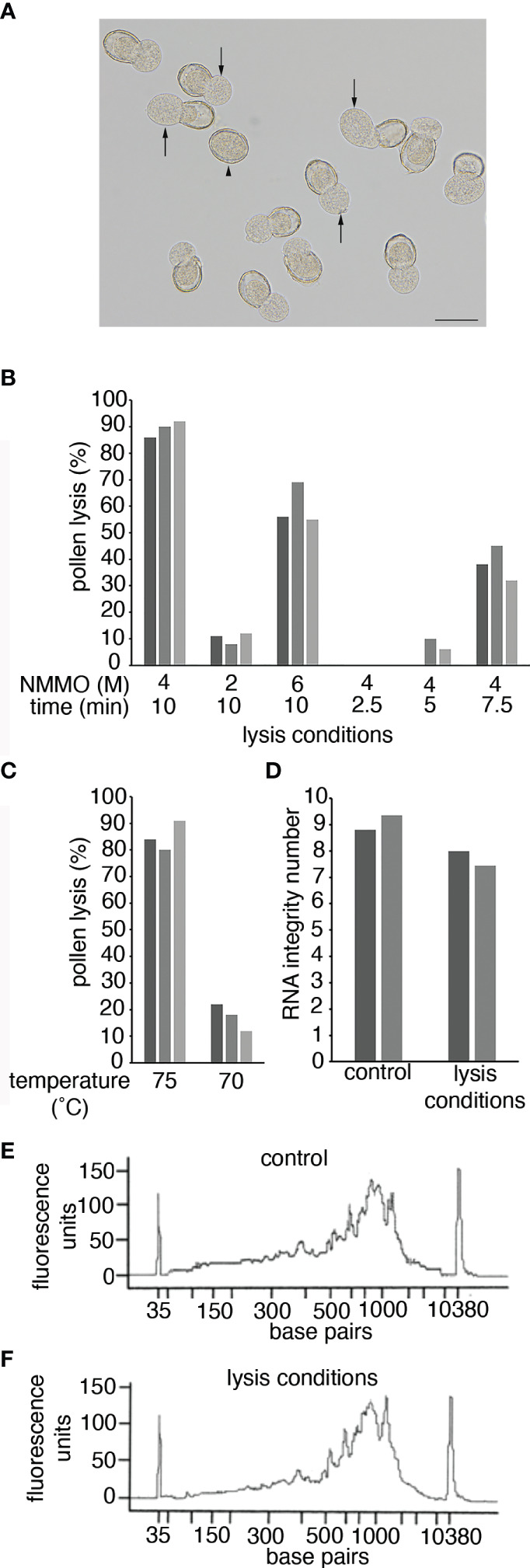
Pollen lysis using NMMO. **(A)** Pollen from a -1 Arabidopsis bud after incubation in 4 M NMMO at 75°C for 10 min. Arrows indicate where the sporoplast is extruded from the pollen wall in a lysed pollen grain. The arrowhead indicates an unlysed pollen grain. Scale bar = 20 μm. **(B, C)** Pollen lysis efficiency. Pollen grains from anthers from -1 Arabidopsis buds were incubated on a microscope slide under different conditions. Pollen was incubated at 75°C at different NMMO concentrations and for different times **(B)** or in 4 M NMMO for 10 min at different temperatures **(C)**. To determine lysis efficiency 100 Arabidopsis pollen grains were observed for sporoplast extrusion, as shown in **(A)**. The experiment was repeated on three occasions with each experiment represented by a separate bar. **(D)** The impact of pollen-lysis conditions on RNA quality. RNA Integrity of two Arabidopsis leaf RNA samples (separate bars) with and without exposure to pollen lysis conditions (4 M NMMO at 75°C for 10 min). **(E, F)** The impact of pollen lysis conditions on cDNA synthesis. The fragment size distribution of cDNA libraries prepared from a kiwifruit leaf RNA sample incubated on ice (control; **E**) or exposed to pollen lysis conditions **(F)**.

Previous transcriptional analyses of pollen have generally relied on mechanical disruption (grinding in liquid nitrogen) to lyse the pollen (Honys and Twell, 2003; Honys and Twell, 2004; Klodová et al., 2022). However, this requires milligram quantities of pollen and is therefore not suitable for pollen from a single anther, or even a single plant. An alternative approach would be to use enzymes to digest the pollen cell wall similar to protoplast preparation in Arabidopsis (Mathur et al., 1995), but this requires long incubation times at modest temperatures that would likely lead to major transcriptional changes in the pollen. We therefore decided to trial chemical lysis using 4-Methylmorpholine *N*-oxide monohydrate (NMMO), a potent solvent for polysaccharides that is used industrially in the Lyocell process to dissolve cellulose fibres (Krässig et al., 2002). Due to the cellulosic nature of the pollen intine (inner wall layer), treatment with NMMO disrupts cell wall integrity, releasing the cellular contents (sporoplast) into solution (**Figure 2A**). NMMO has previously been used to generate sporoplasts in lily but use has been limited due to the loss of cell viability (Loewus et al., 1985).

To assess lysis efficiency of NMMO, pollen isolated from -1 Arabidopsis buds were lysed on heated microscope slides and observed with a dissecting microscope to determine if sporoplasts were released from the pollen wall (**Figure 2A**). Based on previous use of NMMO (Loewus et al., 1985), we decided to use 4 M NMMO at 75°C for 10 min to lyse pollen. Under these conditions, approximately 80% of the pollen was consistently lysed (**Figures 2B, C**). We then tested if the lysis conditions could be further optimised by altering the concentration of NMMO, altering incubation time or lysis temperature (**Figures 2B, C**). All alterations resulted in reduced lysis efficiency, so in further work pollen was lysed with 4 M NMMO at 75°C for 10 min with the pollen immediately vortexed briefly to free partially released sporoplasts. We refer to this treatment as pollen lysis conditions.

As there is no published literature on the use of NMMO in the context of molecular biology, we were concerned the lysis conditions could result in RNA degradation or could impact the performance of enzymes in downstream processes. To determine if this was the case, we tested the impact pollen lysis conditions had directly on RNA quality and cDNA synthesis. We used purified RNA from Arabidopsis or kiwifruit leaves to ensure sufficient RNA quantity and purity and subjected the RNA to pollen lysis conditions. The RNA was then either analyzed using an RNA Bioanalyzer Chip (Arabidopsis) or used for cDNA synthesis (kiwifruit) and analyzed on a HS DNA Bioanalyzer Chip. When the RNA was exposed to the pollen lysis conditions, there was a small reduction in the RNA Integrity Number (**Figure 2D**), suggesting some loss of RNA integrity. However, NMMO was still present in these samples and NMMO fluorescence could be influencing the Bioanalyzer results. Therefore, for the cDNA generated from RNA incubated in pollen lysis conditions NMMO was removed from the cDNA prior to analysis. The results from the cDNA electropherogram suggest that the pollen lysis conditions had a limited impact on the RNA as the size distribution of the cDNA generated from the control RNA and the RNA exposed to pollen lysis conditions were similar (**Figures 2E, F**). Further, we found that NMMO may provide a protective effect, as treatment of RNA at 75°C for 10 min without NMMO lead to a substantial decrease in RNA integrity and dramatic changes in the cDNA fragment distribution (**Supplementary Figure 1**). The successful generation of cDNA also indicated that the presence of NMMO does not prevent molecular biological techniques from being used on lysed pollen (i.e., it does not inhibit enzymic reactions). We therefore concluded NMMO lysis was suitable to obtain a pollen lysate for the preparation of RNA-Seq libraries.

### Transcriptional analysis of pollen at three stages of development from Arabidopsis

We used the developed pollen isolation protocol to perform RNA-Seq on three different developmental stages of pollen from Arabidopsis. We isolated pollen from one medial anther from each bud from inflorescences of different plants and determined the developmental stage. This enabled us to select four biological replicates with each replicate close to the same developmental stage. The stages selected were; uninucleate microspores that had recently been released from tetrads (UNM; from buds -11 or -12), late bicellular pollen where the generative cell had elongated (and in a few cases divided) and the following inflorescence contained tricellular pollen (BCP; from ~bud -4) and late tricellular pollen taken from the most mature unopened flower (TCP; from bud -1), as shown in **Figures 1D–F**.

Pollen lysates were made for each Arabidopsis pollen sample as described above. Each lysate likely contains only a small amount of mRNA (considering the number of cells lysed). We therefore decided to generate RNA-Seq libraries using a modified version of the SmartSeq2 (Switch Mechanism At the 5’ end of RNA Templates version 2) that is designed for low input RNA (Macaulay et al., 2016; Tantirigama et al., 2016). This protocol generates amplified cDNA (acDNA) libraries from a small quantity of polyadenylated RNA. SmartSeq2 relies on the terminal transferase activity of the Moloney Murine Leukemia Virus (MMLV) Reverse Transcriptase, which adds several additional nucleotides to the 3’ end of a newly synthesised cDNA strand. These bases provide the site to which template switching oligonucleotides (TSOs) bind. Upon pairing the TSO to the newly added 3’ bases the reverse transcriptase continues cDNA synthesis, “switching” from the RNA as the template to the TSO. The 3’ end of the cDNA is also tagged with oligo(dT)-containing primers. The final product of this protocol thus contains the complete mRNA complement, with universal sequences on both the 3’ and 5’ ends. This makes it possible to universally amplify the entire full-length coding transcriptome independent of sequence composition resulting in acDNA. This is then followed by tagmentation and library preparation.

As the presence of NMMO did not greatly impact cDNA synthesis (**Figures 2E, F**), we initially trialed library preparation and sequencing on acDNA directly synthesised from Arabidopsis pollen lysate. Approximately 7.5 to 11 million reads were obtained for each Arabidopsis direct sample (**Supplementary Table 1**). As a preliminary analysis, we mapped reads to the Arabidopsis genome to determine the distribution of genomic features and performed principal component analysis (PCA) and hierarchical clustering (**Supplementary Figures 2A–C**). While a considerable proportion of the reads mapped to exons (40-66%), the relatively high number of reads mapping to intergenic regions (15-27%), along with a wide scattered distribution in the PCA plot and a lack of stage-specific clusters, suggested some amplification from genomic DNA (gDNA) may have occurred. As NMMO alters hydrogen bonding (Krässig et al., 2002), it is possible the presence of NMMO enhances non-specific binding leading to primers annealing to gDNA during the cDNA synthesis or PCR amplification stages.

We therefore added an oligo-dT bead capture step (Macaulay et al., 2016) to enrich for polyadenylated RNA and remove NMMO and gDNA prior to cDNA synthesis. Fresh Arabidopsis pollen lysates were prepared from the same stages, libraries prepared and the sequencing repeated. 9-23 million reads were obtained for each Arabidopsis enriched sample (**Supplementary Table 1**). Analysis of the data in the same manner revealed that the inclusion of the oligo-dT bead capture greatly increased the proportion of reads mapping to exons and reduced mapping to intergenic regions by almost three-fold in all stages tested (**Supplementary Figure 2D**). Further, PCA and hierarchical clustering showed clear separation of the samples based on the stage of pollen development (**Supplementary Figures 2E, F**). This separation was also consistent with other studies, with UNM and BCP stages being more similar and separated from TCP by the first principal component (PC1: Klodová et al., 2022). However, we did note a decrease in average mapping rate to the TAIR10 reference genome and an increased number of unmapped reads that were indicated by the STAR alignment report as ‘too short’ (data not shown), suggesting the unmapped reads could not be used for seeding an alignment to the reference genome. This may be a result of RNA degradation due to the increased sample preparation time the oligo-dT bead capture step required. However, we decided that based on the improved exonic mapping statistics and sample replicability, the benefits of the protocol modifications outweighed the negatives. Therefore, we continued analyzing the data obtained with the RNA enrichment step and refer to these as single anther (SA) samples.

**Figure 3:**
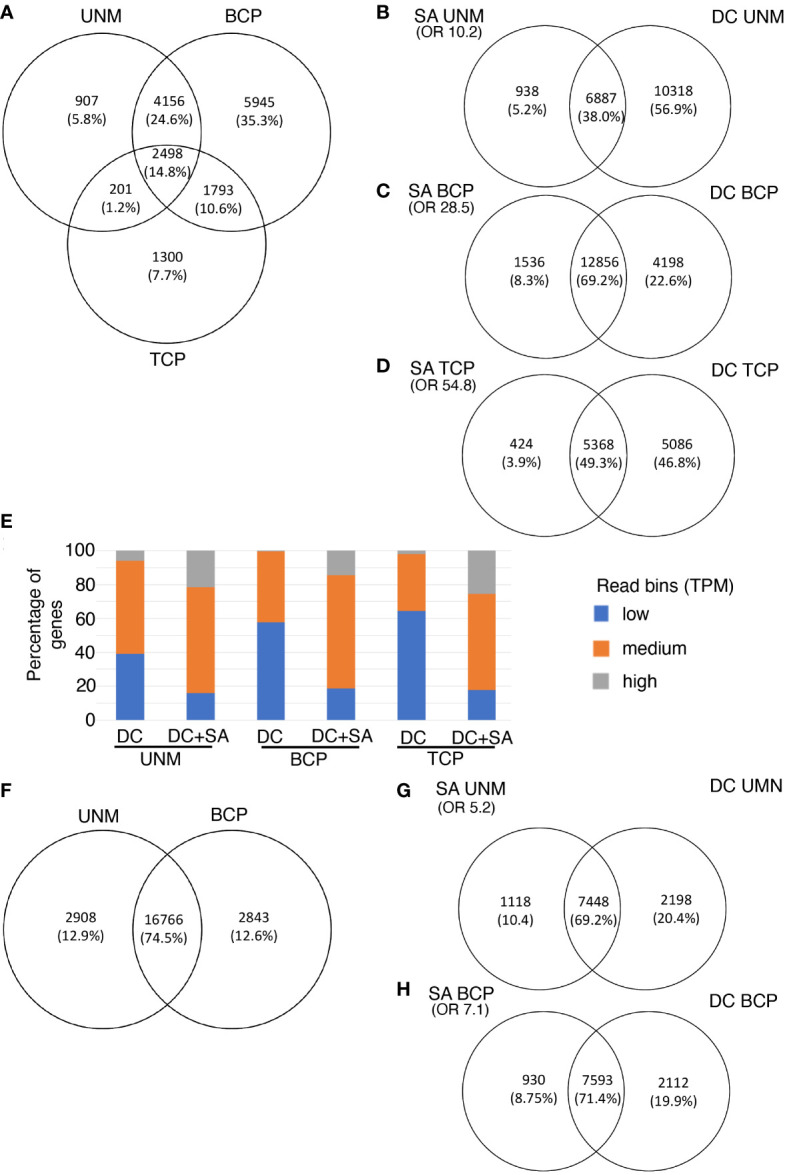
Single anther RNA-Seq for Arabiopsis **(A–E)** and kiwifruit **(F–H)**. **(A–E)** Genes expressed in three stages of pollen development from Arabidopsis **(A)** Venn diagram showing the number of genes and overlap of genes detected by the single anther (SA) RNA-Seq method at three developmental stages. **(B–D)** Overlap of genes at each stage of Arabidopsis pollen development as detected by SA RNA-Seq and density centrifugation (DC) RNA-seq protocols. **(E)** Expression level of genes detected only in the DC data (right panels B to D) or expressed in both the DC and the SC datasets (DC + SA; overlap panels B to D) at each stage of pollen development in Arabidopsis. Genes have been split into expression bins of low (0-10 TPM), medium (10-100 TPM) or high (>100 TPM). **(F–H)** Two stages of pollen development from kiwifruit. **(F)** Venn diagram showing the number of genes and overlap of genes detected by the single anther (SA) RNA-Seq method for kiwifruit. **(G, H)** Overlap of genes at each stage of development as detected by kiwifruit SA RNA-Seq and density centrifugation (DC) RNA-seq protocols. Only genes with reciprocal blast hits (RBHs) between the Arabidopsis and kiwifruit genomes are included. OR, Odds Ratio as determined by the Fisher’s exact test. An OR close to 1 represents means no association between two lists and the higher the value the stronger the association. UNM, uninucleate microspores; BCP, bicellular pollen; TCP, tricellular pollen.

Expression values in transcripts per million (TPM) were calculated and genes defined as expressed if they had a TPM value of 2 or more in at least two of the biological replicates for the stage (**Supplementary Tables 2**-**4**). Overall, 16863 genes were considered expressed in Arabidopsis pollen with 7825 genes at the UNM stage, 14392 at the BCP stage and 5792 at the TCP stage (**Figure 3A**). 2498 genes were expressed at all three stages, with a greater overlap between UNM and BCP stages (4156 genes) than the BCP and TCP stages (1793 genes).

We compared the list of expressed genes in the Arabidopsis SA samples with data from EVOREPRO consortium (Julca et al., 2021; Klodová et al., 2022) where pollen developmental stages were prepared in bulk using density gradient centrifugation (referred to as density centrifugation (DC) samples). The majority (over 90%) of genes expressed in the SA samples were also considered expressed in the DC samples at the corresponding developmental stage (**Figures 3B–D**). The overlap of genes expressed at each stage in the SA and DC pollen datasets was higher than that expected by chance (Fisher’s exact test, shown as an Odds Ratio in **Figures 3B–D**) and is not solely due to highly-expressed constitutive genes [based on higher Odds Ratio values between the two pollen samples than between the DC pollen and a non-pollen (leaf) sample (Berkowitz et al., 2021; **Supplementary Table 5**)]. This confirmed that pollen-expressed genes are being detected in the SA RNA-Seq method.

Many genes were detected in the DC samples that were not detected in the Arabidopsis SA samples, especially at the UNM stage (**Figures 3B–D**). When the expressed genes in both data sets were split into bins of low (TPM 0-10), medium (TPM 10-100) or high (TPM >100) expression, the proportion of highly expressed genes was similar in the SA and DC samples at the UNM and BCP stages, however there were less genes with medium and low expression values in the SA data (**Supplementary Figure 3**). The reduction in the number of genes with low expression values suggests that the low RNA input SA RNA-Seq method may have reduced sensitivity compared to the high-input DC method. We therefore refined our analysis to compare the expression level of the genes detected in the DC and SA data with those detected in the DC data only (**Figure 3E**). The DC-only category contained a higher proportion of the genes in the low expression bin while the overlap (DC and SA expressed) contains a higher proportion of genes in the medium and high expression bins. There were few genes that were highly expressed in the DC data that were not also detected using the SA method. This suggests that the SA method has reduced sensitivity, which is not unexpected for a low-input protocol. Additionally, some of the difference could also be explained by the SA data representing a tight developmental stage rather than an enrichment for a developmental window in the DC data. For example, when Klodová et al. (2022) performed GO analysis on genes differentially expressed between DC UNM and DC BCP samples, meiosis-related genes were included in the categories showing higher expression in UNM samples (that may have contained meiotic cells) compared to the BCP stage (where meiotic cells would be unlikely to be present). Thus, some of the genes with low expression in the DC UNM dataset that were not in the SA UNM dataset could be transcripts related to the presence of meiotic cells in the developmental window sampled with the SC analysis.

**Figure 4:**
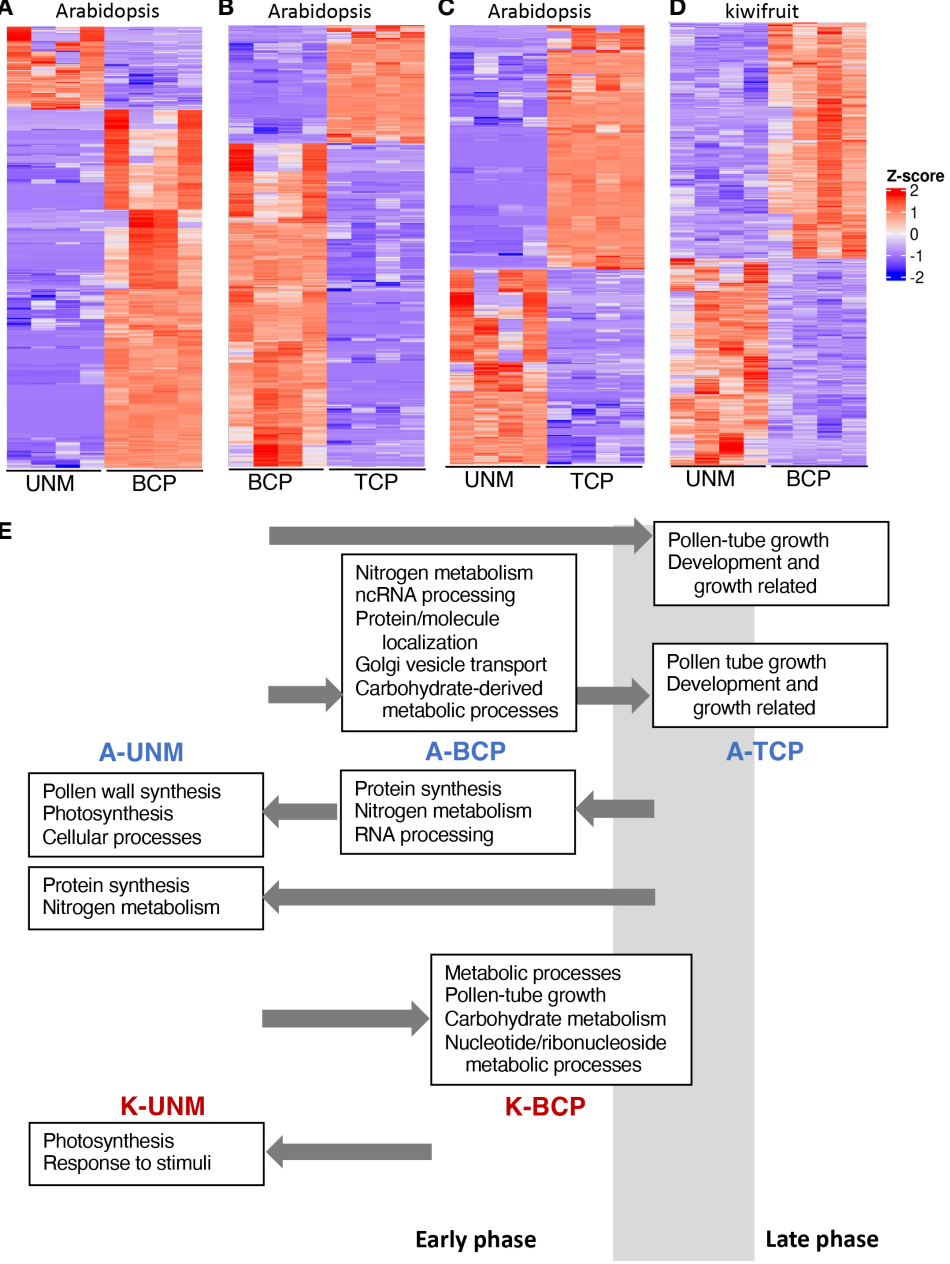
Differential gene expression between pollen stages. **(A–D)** Heatmaps showing differentially expressed genes between the UNM and BCP stages of Arabidopsis **(A)**, the BCP and TCP stages of Arabidopsis **(B)**, the UNM and TCP stages of Arabidopsis **(C)**, and the UNM and BCP stages of kiwifruit **(D)**. Z-score indicates normalized expression level. **(E)** Summary of GO categories enriched at the different stages of pollen development for Arabidopsis (A-UNM, A-BCP and A-TCP) and kiwifruit (K-UNM and K-BCP). Key GO categories are noted in boxes above (from an earlier stage) or below (from a later stage) the stage where they have increased expression and arrows indicate the stage they are being compared to. The estimated timing of the phase transition from the early phase to the late phase in relation to the samples sequenced using the SA RNA-Seq method is indicated by a grey box.

To gain an overview of the changes in gene expression over Arabidopsis pollen development differential gene expression analysis between the stages (UNM-BCP, UNM-TCP and BCP-TCP) was performed (**Figures 4A–C**; **Supplementary Tables 6**-**8**). Consistent with having a greater number of total expressed genes, the BCP stage had a greater number of up-regulated genes compared to either the UNM or TCP stages. We then performed Gene Ontology (GO) enrichment on the genes differentially expressed (separately for the up- and down-regulated genes) between each of the stages (**Figure 4E**; **Supplementary Figures 4**-**6**; **Supplementary Tables 6**-**8**). Consistent with the previously reported phase change between an early phase and late phase (Honys and Twell, 2004; Twell et al., 2006; Klodová et al., 2022), genes with higher expression in the UNM and BCP stages were enriched for GO terms such as translation while pollen-tube growth and developmental related genes were enriched in the TCP stage. As the BCP samples obtained through the SA RNA-seq protocol were from later stages in the developmental window (just before and partially overlapping with PMII), the GO terms observed in this stage in comparison to UNM and TCP indicate that the phase transition may begin during BCP development. For example, Klodová et al. (2022) found that vesicular transport was increased in the tricellular phase, and this GO term is represented in the SA BCP sample. However, the main switch to a pollen-specific transcription program (the differentiation phase), appears to happen in the latter stages of pollen development within the anther, that is during the TCP stage in Arabidopsis. This differs to maize where a sporophyte-to-gametophyte transition has been reported to occur close to PMI (Nelms and Walbot, 2022). The different timing may reflect the length of between the completion of meiosis and PMI, which in maize is ~12 days (Nelms and Walbot, 2022) while in Arabidopsis it is 2 to 3 days (Valuchova et al., 2020).

While the GO analysis was largely similar to other studies, there were a couple of unexpected categories upregulated in the Arabidopsis UNM stage. One unexpected GO category was photosynthesis. However, of the 22 genes expressed in the SA UNM sample contributing to the photosynthesis GO category, 15 were also detected in the UNM and BCP stages in the DC data (**Supplementary Table 9**). Thus, it is likely these genes are expressed in microspores and may relate to plastid/organelle functions not directly related to photosynthesis.

The other unexpected GO categories related to synthesis of the pollen wall, including sporopollenin biosynthesis (**Figure 4E**, **Supplementary Figure 4**). Sporopollenin synthesis proteins have been reported to be located in both tapetal cells and the anther locule during the UNM and BCP stages based on fluorescent fusion proteins (Wang et al., 2018). A lack of fluorescence in microspores lead Wang et al. (2018) to conclude that the sporopollenin synthesis genes were being transcribed and translated within the tapetal cells and released into the anther locule. The presence of the mRNA for these genes in the SA data could therefore reflect microspore expression, or mRNA contamination from tapetal cells even though there was no visible contamination from anther cells or cellular debris after the wash with mannitol.

To test if there are non-microspore derived mRNA transcripts in the washed pellet, we performed reverse-transcriptase quantitative PCR (RT-qPCR) on the supernatant and pellet from the washing steps. We assumed that as the pollen had not been lysed, all transcripts would be from another cells, so used primers for the constitutive *ACT2* gene, along with primers for *LTP12*, a reported tapetal specific gene (Xu et al., 2010). While no PCR product was detected in all samples in the absence of reverse transcriptase, the fluorescence signal in the qPCR reached the cycle threshold (C_t_) after 30 to 35 cycles for the two genes in the supernatant following each of three spins as well as the final pellet (**Supplementary Figure 7**). As the primers included the tapetal gene *LTP12*, this suggests that while washing removes some mRNA, there are still tapetal-derived mRNA transcripts in the isolated pollen pellet. Furthermore, these genes were only detected at low levels in the DC pollen samples (data not shown).

These results suggested that there may be anther-derived transcripts in the pollen pellet, mainly in the UNM developmental stage when the tapetum is highly transcriptionally active. We attempted to mitigate this by washing the pollen, which removed any visible contamination, but may not have removed small molecules. The wash steps were designed to be short to avoid substantial activation of gene expression not found in pollen within the anther, and to also protect the pollen from osmotic stress by using the osmo-protectant mannitol. This combination may have meant that transcripts remained associated with the pollen wall during the washes. It may be possible to either reduce the concentration of mannitol, or complete washes in a different buffer, which may release a greater proportion of the mRNA when pollen in resuspended during the washes, reducing the number anther-derived transcripts. The presence of anther-derived transcripts does not prevent the use of the SA RNA-Seq method for the UNM stage as a tool to survey or compare gene expression, but adequate caution is recommended, and further work would be required to confirm in which cell type a gene is expressed.

### Transcriptional analysis of pollen at two stages of development from kiwifruit

Unlike Arabidopsis that releases pollen in the tricellular state, kiwifruit pollen is bicellular when released (Matsunaga et al., 1996). While pollen mitosis II does not occur in the kiwifruit pollen before anthesis, it is expected that microspores and the vegetative cell will have a similar transcriptional profile to the Arabidopsis pollen; that is an early growth phase and a late phase enriched in transcripts encoding proteins involved in pollen germination and pollen-tube growth shortly before anthesis. To confirm that the SA RNA-Seq protocol can be applied to a different species, and to explore the transcriptional profile of kiwifruit pollen, pollen samples were isolated from kiwifruit buds and samples confirmed by microscopy to be at the UNM and BCP developmental stages. As done in Arabidopsis, four biological replicates were generated for each developmental stage. The SA RNA-Seq protocol was performed and approximately 5-16 million reads were generated for each kiwifruit SA sample (**Supplementary Table 1**). Preliminary analyses using PCA and hierarchical clustering indicated the two developmental stages clearly separated from one another (**Supplementary Figures 8A, B**). There is, however, a greater spread of the samples at each stage compared to the Arabidopsis SA samples. This is potentially a reflection of a greater developmental spread across the samples as the sizes of the buds did not change greatly during pollen development, making it difficult to select anthers at the same developmental stage in kiwifruit (**Figures 1I, J**).

The eight kiwifruit pollen RNA-seq libraries were then aligned to the male *Actinidia chinensis* var. c*hinensis* acc “Russell” genome assembly (Tahir et al., 2022). Sequencing data mapped to the assembly with a rate of approximately 90% for all samples (**Supplementary Table 1**). This mapping rate was higher than for the Arabidopsis samples, likely due to kiwifruit anthers containing more pollen grains, and thus having a higher amount of input RNA for the library preparation. Using the same criteria for expression as for Arabidopsis, a total of 22517 genes were expressed in kiwifruit pollen, representing ~65% of the annotated genes which is similar to the ~62% of the Arabidopsis annotated genes expressed in the Arabidopsis SA data. This included 19674 and 19609 genes expressed in the kiwifruit UNM and BCP stages respectively, and 16766 genes were expressed in both stages (**Figure 3F**; **Supplementary Tables 10**, **11**).

No transcriptomic data for isolated pollen prepared using another method is available for kiwifruit. Therefore, to generate a common proxy for comparison of expression data from kiwifruit pollen to that of Arabidopsis, a Reciprocal BLAST Hit (RBH) search was done for the *A. chinensis* proteome against the Arabidopsis TAIR10.1 peptide assembly. A total of 12465 RBHs were identified between the *A. chinensis* and Arabidopsis assemblies, of which 9568 were expressed in either UNM or BCP in kiwifruit pollen (**Supplementary Tables 10**, **11**). When comparing the list of expressed RBHs in kiwifruit SA samples to the expressed RBHs from the Arabidopsis DC pollen data, a higher number of kiwifruit RBHs than expected by chance were expressed in the corresponding stage in the Arabidopsis pollen data (**Figures 3G, H**; **Supplementary Table 5**). Similar to the observations made in the SA Arabidopsis data, more genes were identified as exclusively expressed in the DC data, again likely an indication of lower sensitivity for expression detection in SA sequencing libraries. When the kiwifruit bicellular pollen was compared to the Arabidopsis DC TCP samples, the overlap was reduced to 55%, (**Supplementary Figure 8C**) suggesting the kiwifruit BCP stage sampled is more like Arabidopsis BCP pollen than Arabidopsis TCP pollen.

We performed differential gene expression analysis between the two kiwifruit pollen stages, showing 1039 genes with higher expression in the UNM stage and 1196 in the BCP stage (**Figure 4D**, **Supplementary Table 12**). GO enrichment analyses (**Figure 4E**, **Supplementary Figure 9**, **Supplementary Table 12**), showed photosynthesis related genes and tapetum development were upregulated in the kiwifruit UNM stage, similar to the SA Arabidopsis UNM samples. A range of GO terms were enriched in the kiwifruit BCP samples, including some that were also enriched in Arabidopsis BCP stage pollen, such as carbohydrate metabolism (**Figure 4E** and Klodová et al., 2022) and nucleotide metabolism (Klodová et al., 2022) and others enriched in the Arabidopsis TCP stage, including pollen-tube development (**Figure 4E** and Klodová et al., 2022), and ATP metabolic processes (Klodová et al., 2022). Thus, it appears that during the development of pollen in kiwifruit a similar shift from an early to a late phase occurs, and while the BCP samples may be more similar to the Arabidopsis BCP shortly before PMII, are likely entering the late phase.

To shed further light on the development of BCP pollen in kiwifruit and compare this to the BCP and TCP stages of development in Arabidopsis it would be useful to have clearly separated early and late BCP pollen stages from kiwifruit. The SA RNA-Seq protocol would be suitable to achieve this. One approach would utilise the high number of anthers in kiwifruit buds and the robust nature of the buds. A hole or window could be cut within a bud through which some anthers could be removed. Anthers could be analysed by microscopy to determine stages over time. When microspores are observed to be completing PMI, anthers could be harvested, confirmed to contain BCP and used for SA RNA-Seq. The bud could then be left to develop further for several days, and a new window cut on the opposite side to harvest anthers for a late BCP sample. Thus, there is potential for further adaptation of the SA RNA-Seq technique to explore pollen transcriptomics in kiwifruit, and potentially other species with different floral morphology.

## Conclusions

We have developed a protocol to enable RNA-Seq of a precise developmental stage of pollen isolated from a single anther, SA RNA-Seq, and shown it can be used on multiple species. While some care will be required when exploring genes detected as expressed at the UNM stage due to the possibility of anther-derived transcripts, we hope that this method will facilitate other studies into gene expression during pollen development in a range of plants. It offers the advantages that it does not require specialised equipment, provides information on a determined stage of pollen development and does not require a large number of plants meaning it can be used for the analysis of developing pollen from plants exposed to a variety of environmental conditions or a number of first-generation transgenic plants. Using a single anther may also offer an opportunity to harvest anthers from the same bud over a developmental window, depending on the floral morphology. Thus, this protocol may be particularly useful for the preliminary analyses and the generation of hypotheses in both the model plant Arabidopsis as well as other model and crop species.

## Data availability statement

The data presented in the study are deposited in the SRA depository, accession number PRJNA930874.

## Author contributions

LL, SC, SV and LB performed experiments, LL performed bioinformatic analyses, RD assisted in experimental design and work, LL, SP and LB designed the experiments and wrote the manuscript.

## References

[B1] Berkowitz O. Xu Y. Liew L. C. Wang Y. Zhu Y. Hurgobin B.. (2021). RNA-Seq analysis of laser microdissected arabidopsis thaliana leaf epidermis, mesophyll and vasculature defines tissue-specific transcriptional responses to multiple stress treatments. Plant J. 107, 938–955. doi: 10.1111/tpj.15314 33974297

[B2] Bokvaj P. Hafidh S. Honys D. (2015). Transcriptome profiling of male gametophyte development in *Nicotiana tabacum* . Genomics Data 3, 106–111. doi: 10.1016/j.gdata.2014.12.002 26484158 PMC4535457

[B3] Bonnot T. Gillard M. B. Nagel D. H. (2019). A simple protocol for informative of enriched gene ontology terms. Bio-Protocol, e3429. doi: 10.21769/BioProtoc.3429

[B4] Borg M. Brownfield L. Twell D. (2009). Male Gametophyte development: A molecular perspective. J. Exp. Bot. 60, 1465–1478. doi: 10.1093/jxb/ern355 19213812

[B5] Borges F. Gardner R. Lopes T. Calarco J. P. Boavida L. C. Keith R.. (2012). FACS-based purification of arabidopsis microspores, sperm cells and vegetative nuclei. Plant Methods 8, 44. doi: 10.1186/1746-4811-8-44 23075219 PMC3502443

[B6] Brewbaker J. L. (1967). The distribution and phylogenetic significance of binucleate and trinucleate pollen grains in the angiosperms. Am. J. Bot. 54, 1069–1083. doi: 10.2307/2440530

[B7] Chen S. Zhou Y. Chen Y. Gu J. (2018). Fastp: an ultra=fast all-in-one FASTQ preprocessor. Bioinformatics 34, i884–i890. doi: 10.1093/bioinformatics/bty560 30423086 PMC6129281

[B8] Cheng C. Y. Krishnakumar V. Chan A. P. Thibaud-Nissen F. Schobel S. Town C. D. (2017). Araport 11: a complete reannotation of the *Arabidopsis thaliana* reference genome. Plant J. 89, 789–804. doi: 10.1111/tpj.13415 27862469

[B9] Dupl’áková N. Dobrev P. I. Reňák D. Honys D. (2016). Rapid separation of arabidopsis male gametophyte developmental stages using a percoll gradient. Nat. Protoc. 11, 1817–1832. doi: 10.1038/nprot.2016.107 27583643

[B10] Fraser L. G. McNeilage M. A. (2016). Reproductive biology the kiwifruit genome (Switzerland: Springer International Publishing), 65–84. doi: 10.1007/978-3-319-32274-2_6

[B11] García-Alcalde F. Okonechnikov K. Carbonell J. Cruz L. M. Götz S. Tarazona S.. (2012). Qualimap: evaluating next-generation sequencing alignment data. Bioinformatics 28, 2678–2679. doi: 10.1093/bioinformatics/bts503 22914218

[B12] Ge S. X. Jung D. Yao R. (2020). ShinyGO: a graphical gene-set enrichment tool for animals and plants. Bioinformatics 36, 2628–2629. doi: 10.1093/bioinformatics/btz931 31882993 PMC7178415

[B13] Hafidh S. Fíla J. Honys D. (2015). Male Gametophyte development and function in angiosperms: a general concept. Plant Reprod. 29, 31–51. doi: 10.1007/s00497-015-0272-4 26728623

[B14] Heslop-Harrison J. (1987). Pollen germination and pollen-tube growth. Int. Rev. Cytol 107, 1–78. doi: 10.1016/S0074-7696(08)61072-4

[B15] Hobo T. Suwabe K. Aya K. Suzuki G. Yano K. Ishimizu T.. (2008). Various spatiotemporal expression profiles of anther-expressed genes in rice. Plant Cell Physiol. 49, 1417–1428. doi: 10.1093/pcp/pcn128 18776202 PMC2566926

[B16] Honys D. Twell D. (2003). Comparative analysis of the arabidopsis pollen transcriptome. Plant Physiol. 132, 640–652. doi: 10.1104/pp.103.020925 12805594 PMC167004

[B17] Honys D. Twell D. (2004). Transcriptome analysis of haploid male gametophyte development in arabidopsis. Genome Biol. 5, R85. doi: 10.1186/gb-2004-5-11-r85 15535861 PMC545776

[B18] Julca I. Ferrari C. Flores-Tornero M. Proost S. Lindner A. C. Hackenberg D.. (2021). Comparative transcriptomic analysis reveals conserved transcriptional programs underpinning organogenesis and reproduction in land plants. Nat. Plants 7, 1143–1159. doi: 10.1038/s41477-021-00958-2 34253868

[B19] Keller M. SPOT-ITN Consortium Simm S. (2018). The coupling of transcriptome and proteome adaption during development and heat stress response of tomato pollen. BMC Genomics 19, 447. doi: 10.1186/s12864-018-4824-5 29884134 PMC5994098

[B20] Klodová B. Potesil D. Steinbachová L. Michaelidis C. Lindner A. C. Hackenberg D.. (2022). Regulatory dynamics of gene expression in the developing male gametophyte of arabidopsis. Plant Reprod. doi: 10.1007/s00497-022-00452-5 PMC10363097 36282332

[B21] Krässig H. Schurz J. Steadman R. G. Schliefer K. Albrecht W. Mohring M.. (2002). Cellulose (Weinheim: Wiley-VCH: Ullmann's Encyclopedia of Industrial Chemistry). doi: 10.1002/14356007.a05_37

[B22] Liao Y. Smyth G. K. Shi W. (2014). featureCounts: an efficient general purpose program for assigning sequence reads to genomic features. Bioinformatics 30, 923–930. doi: 10.1093/bioinformatics/btt656 24227677

[B23] Loewus F. A. Baldi B. G. Franceschi V. R. Meinert L. D. McCollum J. J. (1985). Pollen sporoplasts: Dissolution of pollen walls. Plant Physiol. 78, 652–654. doi: 10.1007/BF00233637 16664301 PMC1064794

[B24] Macaulay I. C. Teng M. J. Haerty W. Kumar P. Ponting C. P. Voet T. (2016). Separation and parallel sequencing of the genomes and transcriptomes of single cells using G&T-seq. Nat. Protoc. 11, 2081–2103. doi: 10.1038/nprot.2016.138 27685099

[B25] Mathur J. Konez C. Szabados L. (1995). A simple method for isolation, liquid culture, transformation and regeneration of *Arabidopsis thaliana* protoplasts. Plant Cell Rep. 14, 221–226. doi: 10.1007/BF00233637 24190299

[B26] Matsunaga S. Sakai A. Kawano S. Kuroiwa T. (1996). Cytological analysis of the mature pollen of *Actinidia deliciosa* (Kiwifruit). Cytologia 61, 337–341. doi: 10.1508/cytologia.61.337

[B27] McCormick S. (2004). Control of male gametophyte development. Plant Cell 16 (suppl.), S142–S153. doi: 10.1105/tpc.016659 15037731 PMC2643393

[B28] Nelms B. Walbot V. (2022). Gametophyte genome activation occurs at pollen mitosis I in maize. Science 375, 424–429. doi: 10.1126/science.abl7392 35084965

[B29] Oliveros C. J. C. (2015) Venny. an interactive tool for comparing lists with venn's diagrams. Available at: https://bioinfogp.cnb.csic.es/tools/venny/index.html.

[B30] Rutley N. Twell D. (2015). A decade of pollen transcriptomics. Plant Reprod. 28, 73–89. doi: 10.1007/s00497-015-0261-7 25761645 PMC4432081

[B31] Smyth D. R. Bowman J. L. Meyerowitz E. M. (1990). Early flower development in arabidopsis. Plant Cell 2, 755–767. doi: 10.1105/tpc.2.8.755 2152125 PMC159928

[B32] Suwabe K. Suzuki G. Takahashi H. Shiono K. Endo M. Yano K.. (2008). Separated transcriptomes of male gametophyte and tapetum in rice: validity of a laser microdissection (LM) microarray. Plant Cell Physiol. 49, 1407–1416. doi: 10.1093/pcp/pcn124 18755754 PMC2566930

[B33] Tahir J. Crowhurst R. Deroles S. Hilario E. Deng C. Schaffer R.. (2022). First chromosome-scale assembly and deep floral-bud transcriptome of a male kiwifruit. Front. Genet. 16. doi: 10.3389/fgene.2022.852161 PMC9149279 35651931

[B34] Tantirigama M. L. Oswald M. J. Clare A. J. Wicky H. E. Day R. C. Hughes S. M.. (2016). *Fezf2* expression in layer 5 projection neurons of mature mouse motor cortex. J. Comp. Neurol. 524, 829–845. doi: 10.1002/cne.23875 26234885

[B35] Twell D. Oh S-A. Honys D. (2006). Pollen development, a genetic and transcriptomic view. In: Malhó R (ed) The pollen tube, vol 3. Plant cell monographs. (Berlin: Springer), 15–45. doi: 10.1007/7089_042

[B36] Valuchova S. Mikulkova P. Pecinkova J. Klimova J. Krumnikl M. Bainar P.. (2020). Imaging plant germline differentiation within arabidopsis flowers by light sheet microscopy. Elife 9, e52546. doi: 10.7554/eLife.52546 32041682 PMC7012603

[B37] Wang K. Guo Z. L. Zhou W. T. Zhang C. Zhang Z. Y. Lou Y.. (2018). The regulation of sporopollenin biosynthesis genes for rapid pollen wall formation. Plant Physiol. 178, 283–294. doi: 10.1104/pp.18.00219 30018171 PMC6130021

[B38] Wei L. Q. Xu W. Y. Deng Z. Y. Su Z. Xue Y. Wang T. (2010). Genome-scale analysis and comparison of gene expression profiles in developing and germinated pollen in *Oryza sativa* . BMC Plant Biol. 11, 338. doi: 10.1186/1471-2164-11-338 PMC2895629 20507633

[B39] Williams J. H. Taylor M. L. O'Meara B. C. (2014). Repeated evolution of tricellular (and bicellular) pollen. Am. J. Bot. 101, 559–571. doi: 10.3732/ajb.1300423 24663667

[B40] Xu J. Yang C. Yuan Z. Zhang D. Gondwe M. Y. Ding Z.. (2010). The ABORTED MICROSPORES regulatory network is required for postmeiotic male reproductive development in *Arabidopsis thaliana* . Plant Cell 22, 91–107. doi: 10.1105/tpc.109.071803 20118226 PMC2828693

[B41] Zhang C. Guinel F. C. Moffat B. A. (2002). A comparative ultrastructural study of pollen development in arabidopsis thaliana ecotype Columbia and male-sterile mutant *apt1-3* . Protoplasma 219, 59–71. doi: 10.1007/s007090200006 11926068

