# Supplementary material for: RNA-seq analysis of synchronized developing pollen isolated from a single anther
Source: Front Plant Sci. 2023 Apr 3;14:1121570. doi: 10.3389/fpls.2023.1121570 (PMC10106640; doi:10.3389/fpls.2023.1121570)
Supplement: Supplementary file 1 [file DataSheet_1.pdf]

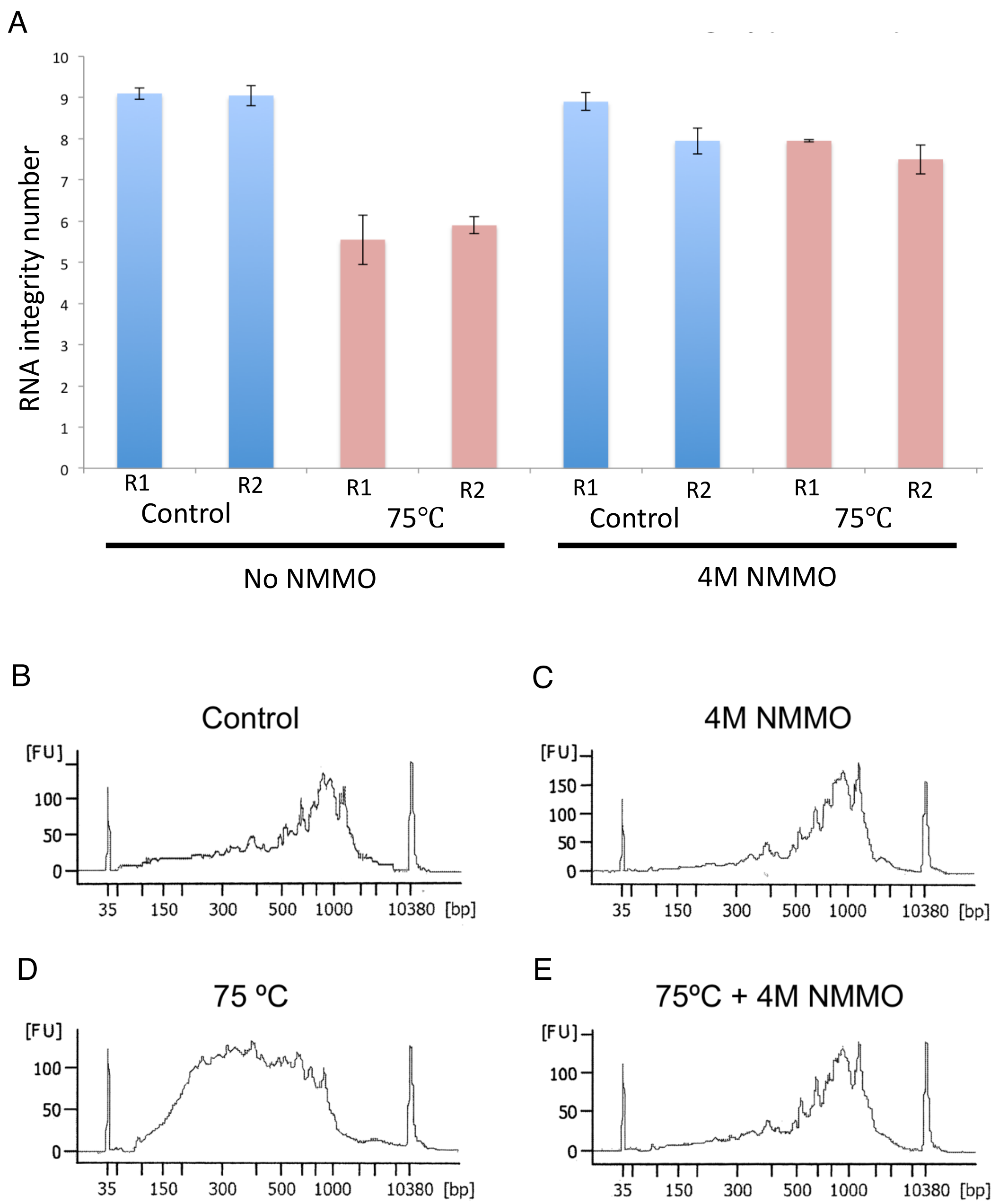

Supplementary Figure 1 NMMO provides a protective effect against heat for RNA. (A) RNA Integrity of two Arabidopsis leaf RNA samples (separate bars) incubated for 10 min on ice (control) or at 75 °C pollen with or without 4M NMMO. (B-E) The fragment size distribution of cDNA libraries prepared from a kiwifruit leaf RNA sample incubated on ice (control) or or at 75 °C pollen with or without 4M NMMO prior to cDNA synthesis.

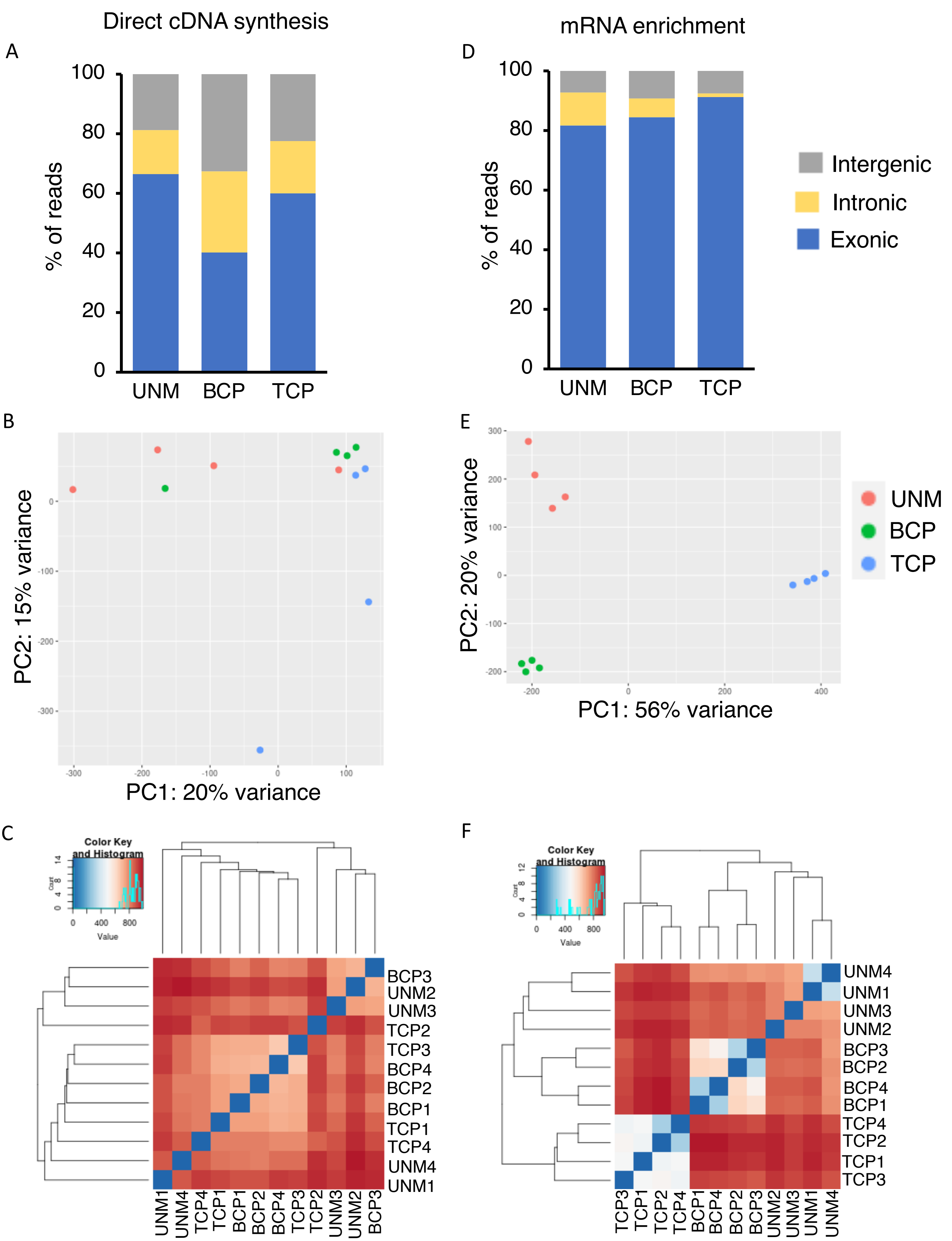

Supplementary Figure 2 Analysis of RNA-Seq libraries from Arabidopsis pollen isolated from a single anther. (**A-C**) Direct cDNA synthesis on pollen lysate. (**D-F**) mRNA enrichment and on-column cDNA synthesis. Each experiment used four biological replicates from three developmental stages; UNM: uninuclear microspores, BCP: bicellular pollen and TCP; tricellular pollen. (A and D) Genomic features of reads mapped to the Arabidopsis genome. Reads were assigned to one of three categories; exon, intron or intergenic. (B and E) Principal component analysis (PCA) plots. (C and F) Hierarchical clustering.

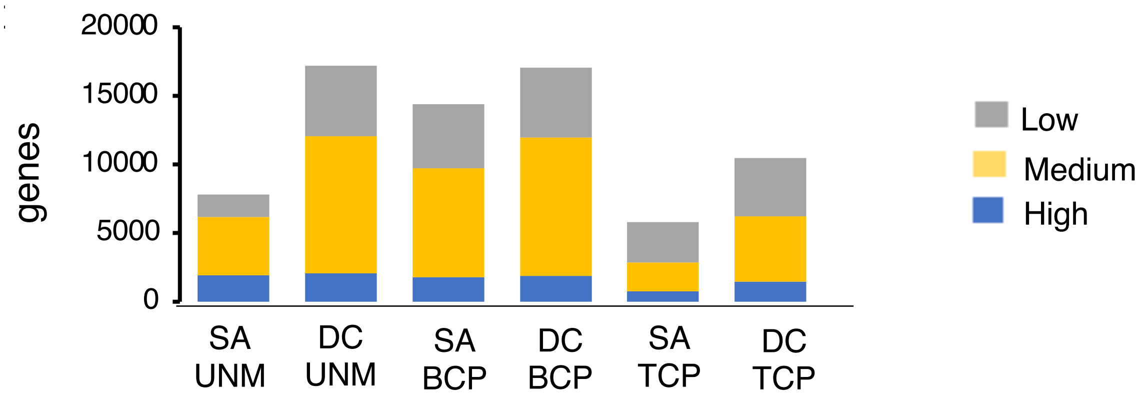

Supplementary Figure 3 Genes from the SA and DC RNA-Seq methods at three stages of pollen development split into expression bins of low (0-10 TPM), medium (10-100 TPM) or high (>100 TPM).

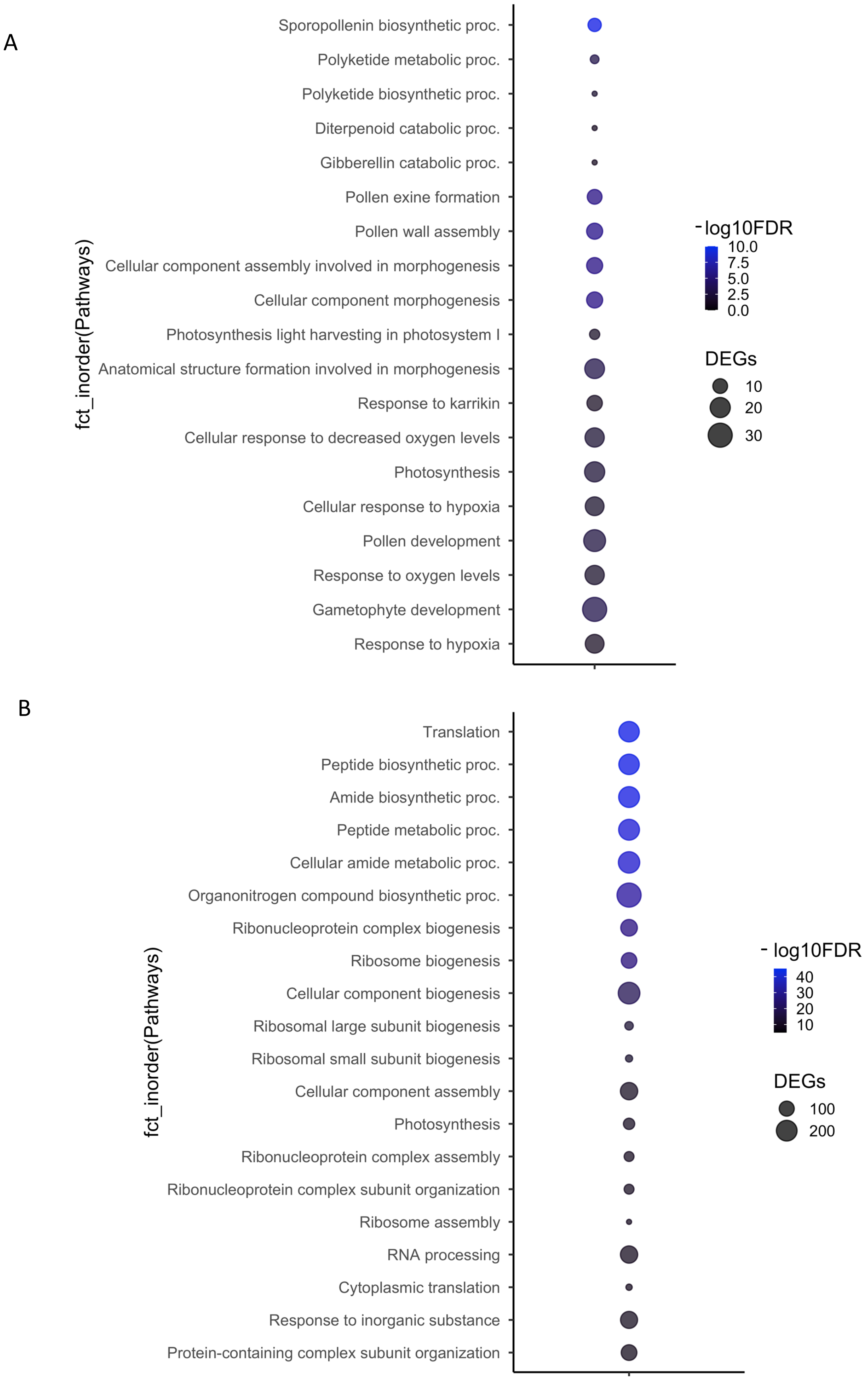

Supplementary Figure 4 GO terms enriched in upregulated genes the SA RNA-Seq Arabidopsis UNM samples compared to the BCP samples (A) and TCP samples (B). The dot size indicates the number of differentially expressed genes associated with the pathways and the dot colour indicates the significance of the enrichment (-log10(FDR-corrected *P*-values)).

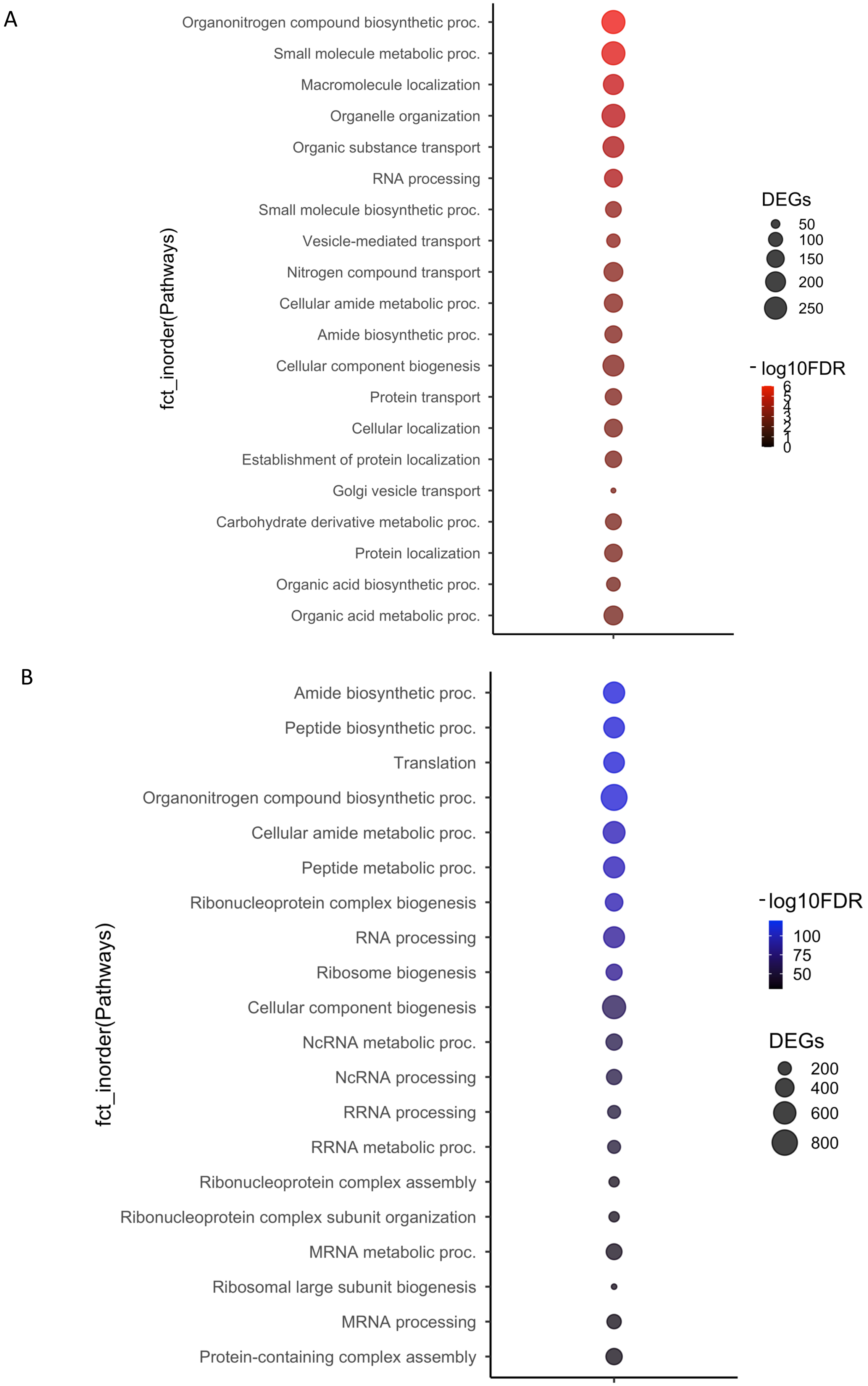

Supplementary Figure 5 GO terms enriched in upregulated genes the SA RNA-Seq Arabidopsis BCP samples compared to the UNM samples (A) and TCP samples (B). The dot size indicates the number of differentially expressed genes associated with the pathways and the dot colour indicates the significance of the enrichment (-log10(FDR-corrected *P*-values)).

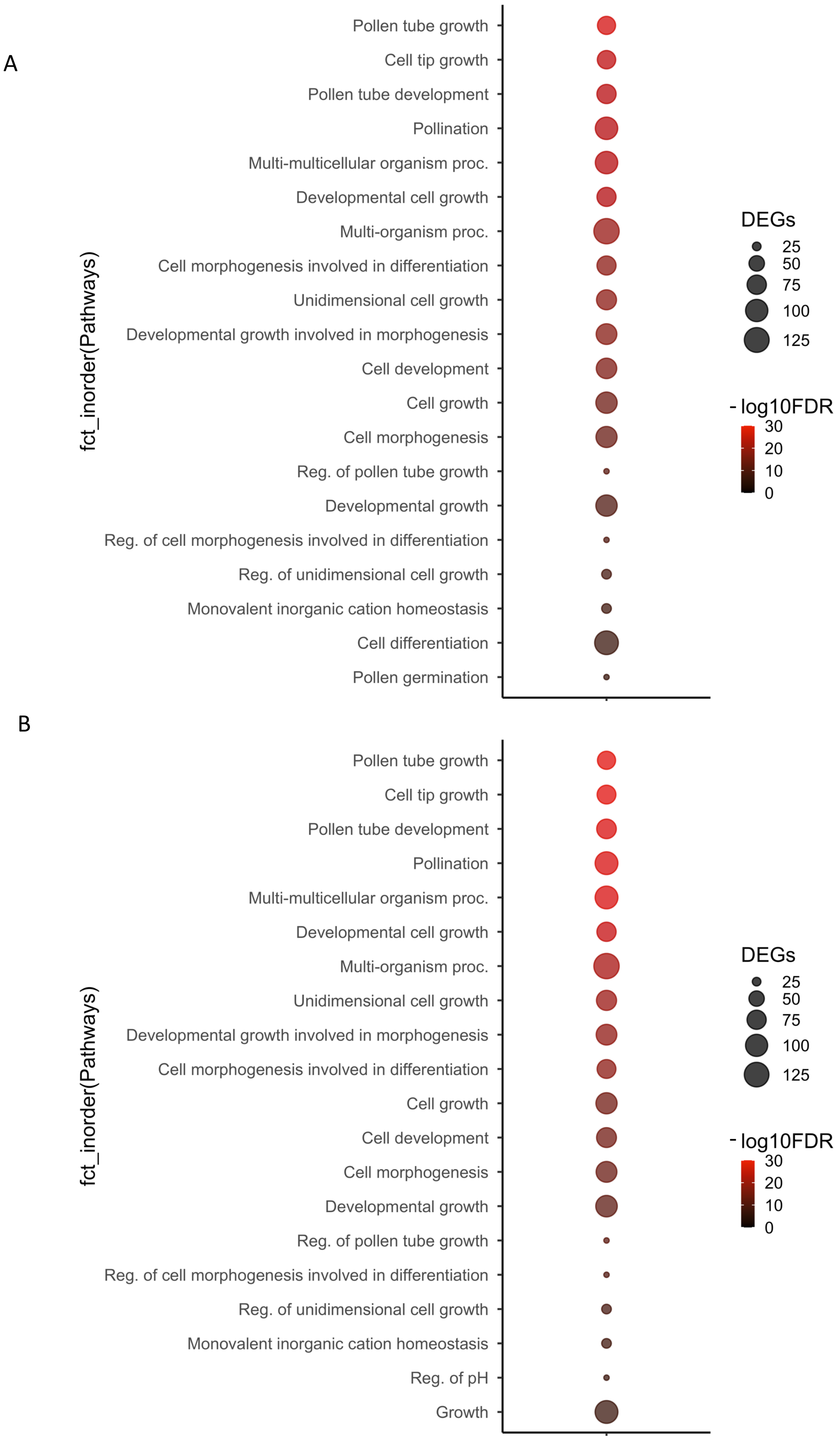

Supplementary Figure 6 GO terms enriched in upregulated genes the SA RNA-Seq Arabidopsis TCP samples compared to the UNM samples (A) and BCP samples (B). The dot size indicates the number of differentially expressed genes associated with the pathways and the dot colour indicates the significance of the enrichment (-log10(FDR-corrected *P*-values)).

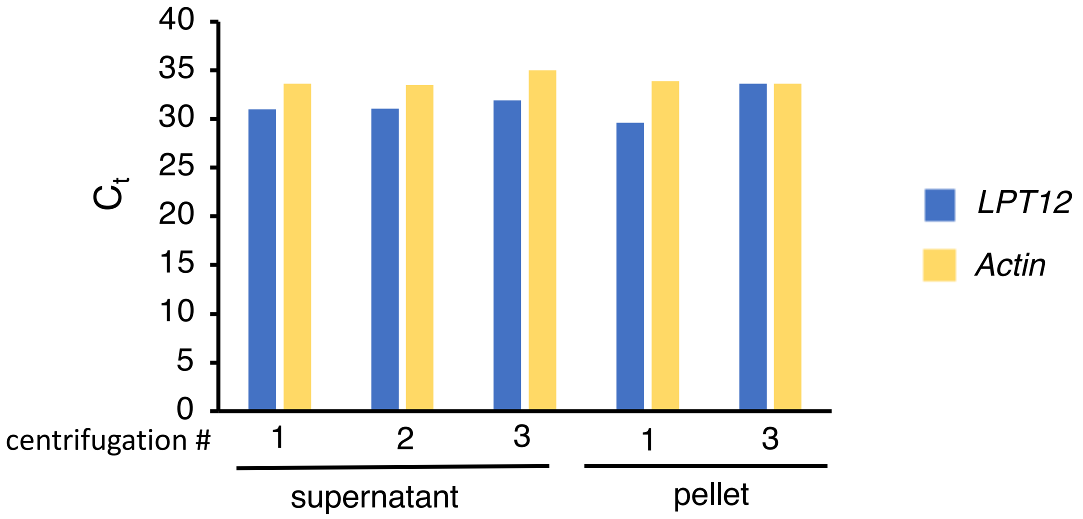

Supplementary Figure 7 Detection of mRNA transcripts in isolated pollen samples. Pollen from a -9 bud were isolated. One sample was centrifuged once to give supernatant 1 and pellet 1. Another pollen sample was centrifuged three times with the supernatants from the second and third centrifugation and the final pellet frozen separately giving samples supernatant 2, supernatant 3 and pellet 3. Each sample was split into + and –RT samples and the cycle threshold (C_t) measured in a quantitative PCR using primers for the gene *LTP12* and *Actin 2*. No product was detected in all -RT samples.

A

B

C

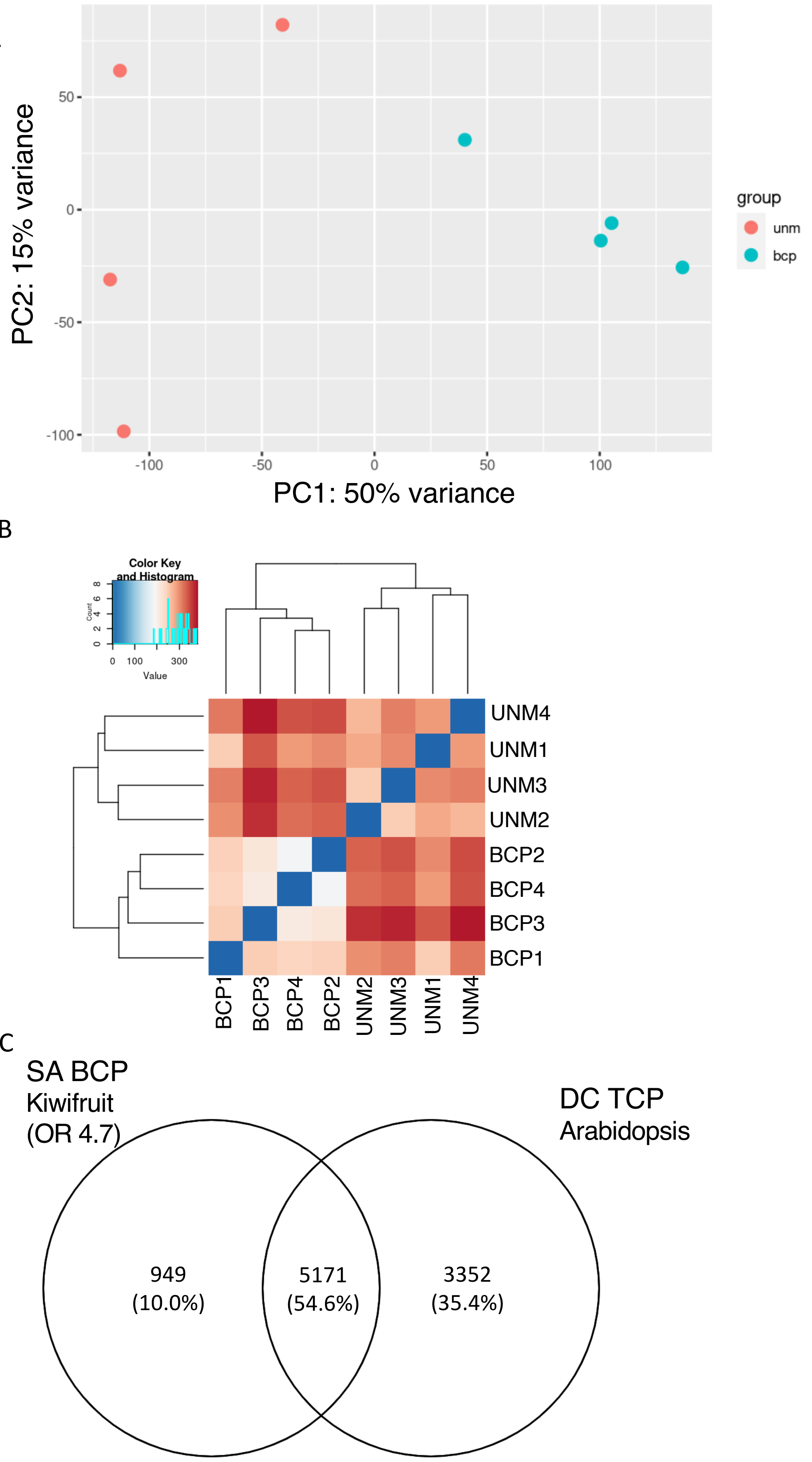

Supplementary Figure 8 Analysis of RNA-Seq libraries from kiwifruit pollen isolated from a single anther with mRNA enrichment and on-column cDNA synthesis. Two stages with four biological replicates were sequenced; UNM: uninuclear microspores, BCP: bicellular pollen (A) Principle component analysis (PCA) plots. (B) Hierarchical clustering. (C) Comparison of the kiwifruit genes expressed at the BCP stage and Arabidopsis genes expressed at the TCP in the DC RNA-Seq data. OR = Odds Ratio (Fisher's exact test; 1 represents means no association between two lists and the higher the value the stronger the association).

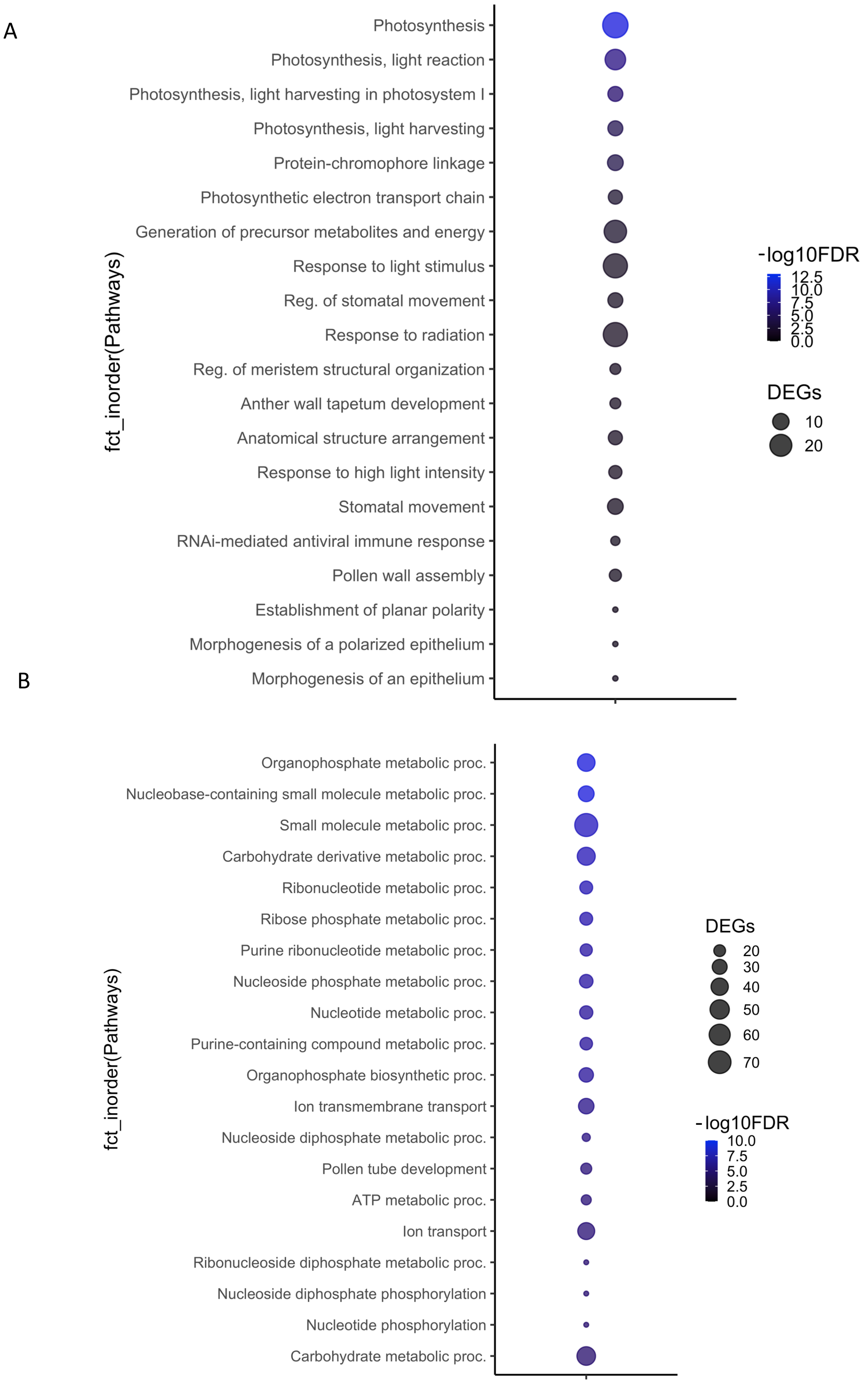

Supplementary Figure 9 GO terms enriched in upregulated genes the SA RNA-Seq kiwifruit UNM sample (A) and BCP sample (B). The dot size indicates the number of differentially expressed genes associated with the pathways and the dot colour indicates the significance of the enrichment (-log10(FDR-corrected *P*-values)).
